# Redox-Regulated Adaptation of Streptococcus oligofermentans to Hydrogen Peroxide Stress

**DOI:** 10.1128/mSystems.00006-20

**Published:** 2020-03-17

**Authors:** Huichun Tong, Yuzhu Dong, Xinhui Wang, Qingqing Hu, Fan Yang, Meiqi Yi, Haiteng Deng, Xiuzhu Dong

**Affiliations:** a State Key Laboratory of Microbial Resources, Institute of Microbiology, Chinese Academy of Sciences, Beijing, China; b University of Chinese Academy of Sciences, Beijing, China; c MOE Key Laboratory of Bioinformatics, School of Life Sciences, Tsinghua University, Beijing, China; University of Wisconsin—Madison

**Keywords:** *Streptococcus*, cysteine oxidation, hydrogen peroxide, posttranslational regulation, redox signaling, transcriptional regulation

## Abstract

Preexposure to a low concentration of H_2_O_2_ significantly increases the survivability of catalase-negative streptococci in the presence of a higher concentration of H_2_O_2_. However, the mechanisms of this adaptation remain unknown. Here, using a redox proteomics assay, we identified 57 and 35 cysteine-oxidized proteins in Streptococcus oligofermentans bacteria that were anaerobically cultured and then pulsed with 40 μM H_2_O_2_ and that were statically grown in a 40-ml culture, respectively. The oxidized proteins included the peroxide-responsive repressor PerR, the manganese uptake repressor MntR, thioredoxin system proteins Trx and Tpx, and most glycolytic proteins. Cysteine oxidations of these proteins were verified through redox Western blotting, immunoprecipitation, and liquid chromatography-tandem mass spectrometry assays. In particular, Zn^2+^-coordinated Cys139 and Cys142 mutations eliminated the H_2_O_2_ oxidation of PerR, and inductively coupled plasma mass spectrometry detected significantly decreased amounts of Zn^2+^ in H_2_O_2_-treated PerR, demonstrating that cysteine oxidation results in Zn^2+^ loss. An electrophoretic mobility shift assay (EMSA) determined that the DNA binding of Mn^2+^-bound PerR protein (PerR:Zn,Mn) was abolished by H_2_O_2_ treatment but was restored by dithiothreitol reduction, verifying that H_2_O_2_ inactivates streptococcal PerR:Zn,Mn through cysteine oxidation, analogous to the findings for MntR. Quantitative PCR and EMSA demonstrated that *tpx*, *mntA*, *mntR*, and *dpr* belonged to the PerR regulons but that only *dpr* was directly regulated by PerR; *mntA* was also controlled by MntR. Deletion of *mntR* significantly reduced the low-H_2_O_2_-concentration-induced adaptation of S. oligofermentans to a higher H_2_O_2_ concentration, while the absence of PerR completely abolished the self-protection. Therefore, a low H_2_O_2_ concentration resulted in the cysteine-reversible oxidations of PerR and MntR to derepress their regulons, which function in cellular metal and redox homeostasis and which endow streptococci with the antioxidative capability. This work reveals a novel Cys redox-based H_2_O_2_ defense strategy employed by catalase-negative streptococci in Mn^2+^-rich cellular environments.

**IMPORTANCE** The catalase-negative streptococci produce as well as tolerate high levels of H_2_O_2_. This work reports the molecular mechanisms of low-H_2_O_2_-concentration-induced adaptation to higher H_2_O_2_ stress in a *Streptococcus* species, in which the peroxide-responsive repressor PerR and its redox regulons play the major role. Distinct from the Bacillus subtilis PerR, which is inactivated by H_2_O_2_ through histidine oxidation by the Fe^2+^-triggered Fenton reaction, the streptococcal PerR is inactivated by H_2_O_2_ oxidation of the structural Zn^2+^ binding cysteine residues and thus derepresses the expression of genes defending against oxidative stress. The reversible cysteine oxidation could provide flexibility for PerR regulation in streptococci, and the mechanism might be widely used by lactic acid bacteria, including pathogenic streptococci, containing high levels of cellular manganese, in coping with oxidative stress. The adaptation mechanism could also be applied in oral hygiene by facilitating the fitness and adaptability of the oral commensal streptococci to suppress the pathogens.

## Abstract (precis)

The catalase-negative streptococci produce as well as tolerate high levels of H_2_O_2_. This work reports the molecular mechanisms of low-H_2_O_2_-concentration-induced adaptation to higher H_2_O_2_ stress in a *Streptococcus* species, in which the peroxide-responsive repressor PerR and its redox regulons play the major role. Distinct from the Bacillus subtilis PerR, which is inactivated by H_2_O_2_ through histidine oxidation by the Fe^2+^-triggered Fenton reaction, the streptococcal PerR is inactivated by H_2_O_2_ oxidation of the structural Zn^2+^ binding cysteine residues and thus derepresses the expression of genes defending against oxidative stress. The reversible cysteine oxidation could provide flexibility for PerR regulation in streptococci, and the mechanism might be widely used by lactic acid bacteria, including pathogenic streptococci, containing high levels of cellular manganese, in coping with oxidative stress. The adaptation mechanism could also be applied in oral hygiene by facilitating the fitness and adaptability of the oral commensal streptococci to suppress the pathogens.

## INTRODUCTION

Reactive oxygen species (ROS), such as superoxide anions (O_2_^−^), hydrogen peroxide (H_2_O_2_), and hydroxyl radicals (HO·), damage almost all biological macromolecules (1–3). Therefore, organisms have evolved diverse mechanisms to cope with ROS (1–4). Facultatively anaerobic streptococci, such as the human opportunistic pathogen Streptococcus pneumoniae and the oral commensal bacterium Streptococcus oligofermentans, do not encode H_2_O_2_-scavenging catalase and thus accumulate endogenous H_2_O_2_ (5–8). Streptococci are also well-known for surviving in the presence of high concentrations of H_2_O_2_ (6, 9, 10). Previously, we determined that statically grown S. oligofermentans cultures have an approximately 200-fold higher survival rate than cells anaerobically cultured in 10 mM H_2_O_2_ (11). A similar observation has also been reported for S. pneumoniae (8). This suggests that the low levels of H_2_O_2_ that accumulate in statically cultured cells may assist streptococci with resisting the oxidant at higher concentrations. However, the biological basis of this low-H_2_O_2_-concentration-induced adaptation remains unknown.

Bacteria usually use cysteine-based redox reactions to sense H_2_O_2_ and activate the downstream peroxide detoxification pathways (12–14). Escherichia coli OxyR was the first identified archetype of thiol-based redox regulators in bacteria; it is activated by intramolecular thiol-disulfide formation resulting from H_2_O_2_ oxidation and thereby induces expression of the genes involved in defending against oxidative stress (15). Gram-positive bacteria, on the other hand, utilize the peroxide-responsive repressor PerR to sense H_2_O_2_ and derepress the H_2_O_2_ resistance genes (11, 16, 17). PerR, a member of the Fur family of metal-dependent regulators, possesses two metal-binding sites: a regulatory Fe^2+^ or Mn^2+^ binding site consisting of histidine and aspartate residues and a structural Zn^2+^ binding site comprising four cysteine residues (18, 19). The Bacillus subtilis PerR is inactivated by H_2_O_2_ via metal-catalyzed oxidation (MCO) (20). When binding Fe^2+^, PerR is inactivated by Fenton chemistry-generated HO· from H_2_O_2_, which oxidizes the histidine residues. In contrast, the cysteine residues of the B. subtilis PerR that coordinate Zn^2+^ for structural maintenance are somehow inert to H_2_O_2_ (20). Therefore, PerR:Zn,Fe (Fe^2+^-bound PerR) but not PerR:Zn,Mn responds to H_2_O_2_ (17, 19). Makthal et al. (21) also reported that H_2_O_2_ inactivates the recombinant Streptococcus pyogenes PerR:Zn,Fe, suggesting that Fe^2+^-triggered Fenton chemistry could inactivate the streptococcal PerR as well. However, an *in vivo* study demonstrated that the S. pyogenes PerR:Zn,Mn also displays a weaker response to H_2_O_2_ (22). Previously, we found that the S. oligofermentans PerR is inactivated by H_2_O_2_ and derepresses the antioxidative non-heme iron-containing ferritin, *dpr*, and manganese importer *mntABC* genes (11). However, even if grown in Mn^2+^-supplemented medium, H_2_O_2_ still induces the expression of *dpr*. This implies that the streptococcal PerR can be inactivated by mechanisms other than Fe^2+^-triggered Fenton chemistry.

The redox-sensing transcriptional regulators usually respond to H_2_O_2_ challenge through cysteine oxidation (12, 13, 23). Recently, this thiol redox switch-based regulatory mechanism was found to be employed by other transcriptional regulators, such as AgrA in the control of the quorum sensing of Staphylococcus aureus (24) and MntR in the regulation of manganese uptake and the oxidative stress resistance of S. oligofermentans (25). Thiol redox proteomics is a powerful approach for the quantification of oxidative thiol modifications and the identification of physiologically important proteins in oxidative stress resistance (26–28). Using this approach, a number of novel redox-regulated proteins that contribute to the protection of E. coli from H_2_O_2_ stress (29) have been identified. Recently, proteome-wide quantification and characterization of the oxidation-sensitive cysteine residues have determined complex and multilayered oxidative stress responses in pathogenic bacteria, such as Pseudomonas aeruginosa, S. aureus, and S. pneumoniae (8, 30). Therefore, cysteine-containing proteins not only serve as H_2_O_2_-damaged targets but also equip bacteria with the capability to resist H_2_O_2_ stress.

To elucidate the mechanisms underlying low-H_2_O_2_-concentration-induced resistance to high concentrations of H_2_O_2_ in streptococci, we employed physiological, biochemical, genetic, and redox proteomics approaches to investigate the H_2_O_2_-sensitive cysteine-containing proteins that may be involved in H_2_O_2_ adaptation. We determined that cellular H_2_O_2_ levels ranging from 40 to 100 μM protected S. oligofermentans from insult by higher H_2_O_2_ concentrations. Redox proteomics identified cysteine oxidation in the H_2_O_2_-responsive transcriptional regulators PerR and MntR, which regulate antioxidative stress in response to H_2_O_2_, as well as in the thioredoxin system proteins Tpx and Trx, which function in thiol-disulfide homeostasis. Importantly, 40 μM H_2_O_2_ oxidized the Zn^2+^-coordinated cysteine residues and inactivated PerR, thus derepressing its regulons, which function in the thiol redox circuit and metal homeostasis. The high sensitivity of the cysteine residues to H_2_O_2_ enables PerR to sense low levels of H_2_O_2_ and thus protect the catalase-negative species S. oligofermentans from H_2_O_2_ challenge by maneuvering the H_2_O_2_ resistance systems. Moreover, the reversible cysteine oxidation resulting from a low H_2_O_2_ concentration can also endow the streptococcal PerR with flexibility in H_2_O_2_-responsive regulation.

## RESULTS

### Preexposure to a low H_2_O_2_ concentration enables S. oligofermentans to resist higher H_2_O_2_ concentrations.

Previously, we found that aerobically cultured S. oligofermentans exhibits significantly higher resistance to H_2_O_2_ stress than anaerobic cultures (11), suggesting that the endogenous H_2_O_2_ that accumulates in the static culture may protect streptococci from damage in the presence of higher H_2_O_2_ concentrations. To validate this presumption, we deleted both the *pox* and *lox* genes, which encode pyruvate oxidase and lactate oxidase, respectively, the two major H_2_O_2_ producers in S. oligofermentans (5, 6). As expected, when exposed to 20 mM H_2_O_2_, only 0.02% survival was found for *pox lox* mutant cells; in comparison, 30% survival was found for wild-type cells (Table 1).

**TABLE 1:** Prepulsing with a low H_2_O_2_ concentration increases the survival rates of various S. oligofermentans strains in the presence of a higher H_2_O_2_ concentration

Culture	Survival rate (%)^*a*^			
	Wild-type strain	Δ*pox* Δ*lox* mutant	Δ*mntR* mutant	Δ*perR* mutant
Static culture	30 ± 3.27*	0.02 ± 0.01*	ND	ND
Anaerobic culture				
Nonprepulse	0.21 ± 0.07	0.23 ± 0.06	7.94 ± 3.18	75 ± 11
Prepulse with 40 μM H_2_O_2_	77 ± 33	66 ± 12	62 ± 26	86 ± 14
Prepulse with 100 μM H_2_O_2_	46 ± 4	ND	ND	ND

a Strains were grown anaerobically in a 6-ml BHI culture, and then the survival percentages in 10 mM H_2_O_2_ were determined for all strains except for strains labeled with an asterisk, which were statically grown in a 40-ml culture and challenged with 20 mM H_2_O_2_. The survival percentage was calculated by dividing the number of CFU in the H_2_O_2_-treated culture by that in the untreated culture. The experiments were repeated three times with triplicate batch cultures each time. The results are averages ± SD from three independent experiments. ND, not determined.

To verify if the loss of H_2_O_2_ resistance in the *pox lox* mutant was due to the lack of endogenous H_2_O_2_ but not the reduction of acetyl phosphate, which is produced by Pox and which contributes to S. pneumoniae H_2_O_2_ resistance (31), we determined whether a preexposure to a low concentration of H_2_O_2_ could increase the higher H_2_O_2_ resistance of the *pox lox* mutant. The wild-type and *pox lox* mutant strains were anaerobically grown until the optical density at 600 nm (OD_600_) was ∼0.5. One aliquot of the cultures, noted as the prepulse group, was pulsed for 20 min with 40 μM H_2_O_2_ prior to a 10-min challenge with 10 mM H_2_O_2_. Another aliquot, the nonprepulse group, was directly treated with 10 mM H_2_O_2_. Samples not treated with 10 mM H_2_O_2_ were included as controls. Table 1 shows that prepulsing with 40 μM H_2_O_2_ greatly improved the survival of the *pox lox* mutant in the presence of 10 mM H_2_O_2_ to 66%, whereas that for the nonprepulsed group was 0.23%. Similarly, prepulsing with 40 μM H_2_O_2_ increased the survival rate of the wild-type strain to 77%, whereas that for the nonprepulsed group was 0.21%. Moreover, prepulsing with 100 μM H_2_O_2_ also elevated the survival rate of the wild-type strain to 46% in the presence of 10 mM H_2_O_2_ (Table 1). These results confirm that endogenous H_2_O_2_ at low concentrations plays an important role in the protection of *Streptococcus* from insult by a higher H_2_O_2_ concentration.

### Estimation of endogenous H_2_O_2_ levels for self-protection and oxidative stress by HyPer fluorescent protein.

**FIG 1:**
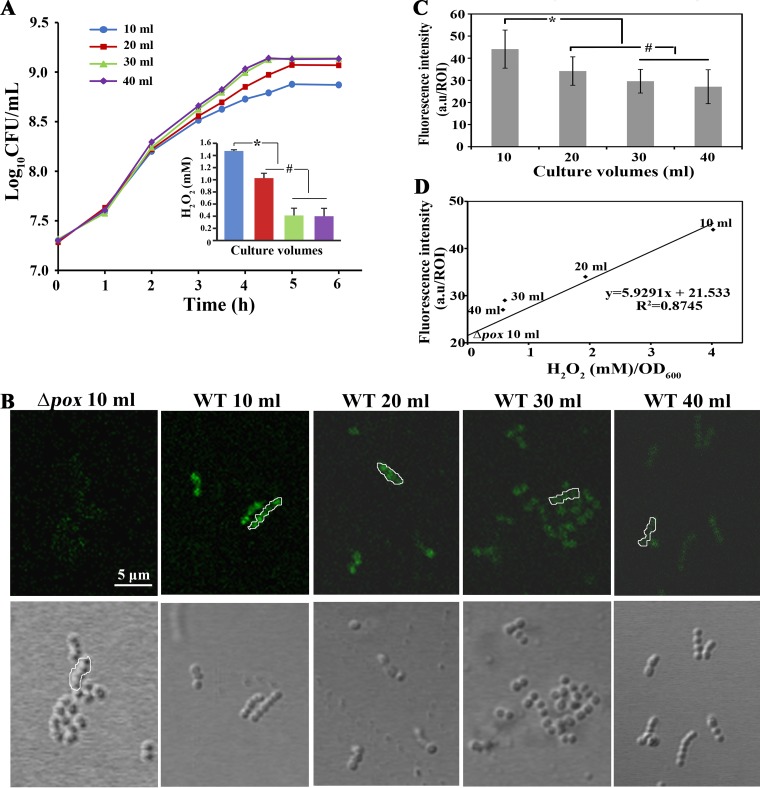
Correlation between growth suppression and the cellular H_2_O_2_ contents of S. oligofermentans. (A) An overnight culture of the HyPer reporter strain WT-HyPer was diluted 1:30 into 10, 20, 30, or 40 ml of BHI broth in 100-ml Erlenmeyer flasks and statically cultured at 37°C. Growth profiles were monitored by counting the numbers of CFU at the indicated time points. (Inset) H_2_O_2_ concentrations in the stationary-phase cultures were determined as described in Materials and Methods and are shown as bar diagrams using the color corresponding to the color of the growth curves for the same culture volumes. Experiments were conducted with three batches of culture and three replicates for each. Averages ± SD from three independent experiments are shown. * and #, the data are significantly different from those determined for 10-ml cultures and those determined for both the 10- and 20-ml cultures, respectively, as verified by one-way analysis of variance followed by Tukey’s *post hoc* test (*P < *0.05). (B) One milliliter of mid-exponential-phase WT-HyPer cells was collected from the cultures for which the results are shown in panel A, washed twice with PBS, and resuspended in 100 μl PBS. After a 30-min air exposure in the dark, HyPer fluorescence was examined using a confocal laser scanning microscope system (Leica model TCS SP8). The Δ*pox*-HyPer cells grown in 10 ml of culture were included as an endogenous H_2_O_2_-negative control. Representative fluorescent and corresponding differential interference contrast (DIC) images from three independent experiments are shown. (C) The HyPer fluorescence intensities of the cells shown in panel B were measured using Leica Application Suite (LAS) AF software. At least five images were captured per sample, and 25 regions of interest (ROI; outlines framed in panel B), each containing 5 cells, were measured for calculation of the average fluorescence intensity of each sample. For images with fluorescence that was too weak, the ROI in the corresponding DIC images was framed, and the fluorescence in the corresponding ROI of the fluorescence image was measured. Average fluorescence intensities were calculated and are expressed in arbitrary units (a.u.) per ROI ± standard deviation. *and #, data are significantly different from those obtained from 10-ml cultures and those determined from both 10- and 20-ml cultures, respectively (*P < *0.05, one-way analysis of variance followed by Tukey’s *post hoc* test). (D) A linear regression curve of the HyPer fluorescence intensities in Δ*pox*-HyPer and WT-HyPer cells plotted against the extracellular H_2_O_2_ concentrations in the corresponding culture volumes.

Given that streptococci accumulate endogenous H_2_O_2_, we used the HyPer fluorescent protein to estimate intracellular H_2_O_2_ levels (32) and compared them with those excreted into the culture. The wild-type (WT) HyPer reporter strain of S. oligofermentans, WT-HyPer (33), was statically grown in 10, 20, 30, and 40 ml of brain heart infusion (BHI) broth in 100-ml flasks, which built an initial O_2_ supply gradient. The growth profiles and the H_2_O_2_ amounts in the cultures were measured. Figure 1A shows that the best growth and the lowest H_2_O_2_ concentration (approximately 400 μM) were measured for the 40-ml culture. In contrast, the poorest growth and the highest H_2_O_2_ level (approximately 1,400 μM) were detected in the 10-ml culture, indicating that larger amounts of H_2_O_2_ are produced by *Streptococcus* with a rich supply of oxygen and thus suppress its growth.

Next, the mid-exponential-phase cells of the WT-HyPer strain from each volume of cultures were visualized under a confocal laser scanning microscope (Leica model TCS SP8), and the HyPer fluorescence intensities were measured as described in Materials and Methods. The Δ*pox*-HyPer mutant, the *pox* deletion mutant carrying the HyPer gene (33), was included as a control from which H_2_O_2_ was absent. Figure 1B and C show that the HyPer fluorescence intensities were inversely proportional to the culture volumes but directly proportional to the H_2_O_2_ concentrations in the cultures, with a good linear regression (*R*^2^ = 0.8745) (Fig. 1D). This indicates that the quantity of H_2_O_2_ in a culture indicates an equivalent amount within the cells.

### Redox proteomics identifies cysteine-oxidized proteins by the low H_2_O_2_ concentration that induces self-protection from oxidative stress.

**FIG 2:**
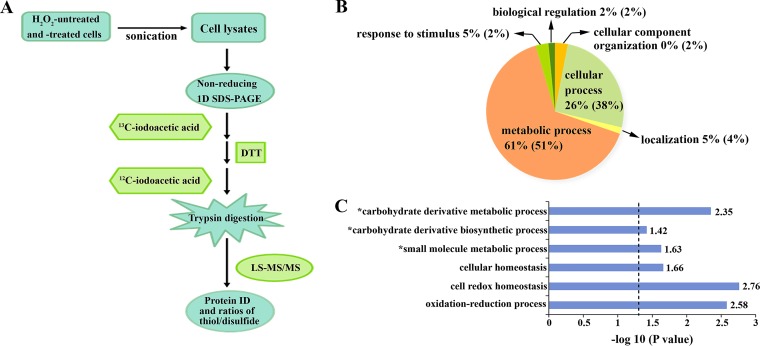
Redox proteomic identification of proteins whose cysteines were oxidized by endogenous or exogenous H_2_O_2_ at levels protecting S. oligofermentans from oxidative stress. (A) Flowchart of redox proteomic analysis, performed using differential alkylation and LC-MS/MS. The free and disulfide-oxidized thiol groups were modified by [^13^C]- and [^12^C]iodoacetic acid, respectively. Detailed experimental procedures are described in Materials and Methods. 1D, one dimensional; ID, identifier. (B) Functional classification of the proteins with cysteine residues reversibly or irreversibly oxidized in the wild-type strain statically grown in a 40-ml culture and the 40 μM H_2_O_2_-pulsed anaerobically grown wild-type strain (percentages in parentheses), based on Gene Ontology (GO) analysis. (C) Overrepresented biological processes associated with cysteine-oxidized proteins were examined using a statistical overrepresentation test on the Gene Ontology Consortium website. The binomial test was used for statistical significance analysis using a *P* value of <0.05 as a cutoff, which is indicated by a dashed line. Asterisks indicate the biological processes enriched in both 40 μM H_2_O_2_-pulsed cells and cells grown in the 40-ml culture. The remaining items were enriched only in cells grown in the 40-ml culture.

To identify the proteins that are sensitive to a low H_2_O_2_ concentration and that might be involved in self-protection from oxidative stress, label-free redox proteomics analysis was performed to identify the cysteine-oxidized proteins in 40 μM H_2_O_2_-pulsed anaerobically grown S. oligofermentans. Proteins were extracted from H_2_O_2_-treated and -untreated cells, and cysteine thiol group oxidations were analyzed using a combination of differential alkylation and liquid chromatography (LC)-tandem mass spectrometry (MS/MS) (28, 34) (Fig. 2A). The representative MS/MS spectra shown in Fig. S1A and B in the supplemental material demonstrated the reliable identification of the cysteine-modified peptides.

10.1128/mSystems.00006-20.1 FIG S1 Representative MS/MS spectra for identification of the redox-sensitive proteins and the cysteine-oxidized and -reduced peptide fragment of PerR, Tpx, and Trx of S. oligofermentans. (A and B) Representative MS/MS spectra of the doubly charged fragment ions at *m/z* 888.9315 (A) and 887.9282 (B), corresponding to peptide fragment AIC^235^EETGNGHVQLFAK from pyruvate kinase with reduced (^13^C-carboxymethylated) and oxidized (^12^C-carboxymethylated) cysteine residues, respectively. (C) The MS/MS spectrum of the doubly charged fragment ion at *m*/*z* 1143.6489, corresponding to the peptide fragment SQMVVYGIC^139^PEC^142^AQQEQVAS from PerR, in which two cysteine residues were oxidized (^12^C carboxymethylated). The spectrum was obtained from a preliminary redox proteomic experiment. (D) MS/MS spectrum of a doubly charged fragment ion at *m/z* 968.5062 for MH_2_^2+^, corresponding to the Tpx peptide fragment VLSIVPSIDTGVC^58^STQTR from H_2_O_2_-untreated cells, in which the cysteine residue was reduced (^13^C carboxymethylated). (E and F) MS/MS spectra of a doubly charged fragment ion at *m/z* 968.5067 and 967.5011 for MH_2_^2+^, corresponding to the Tpx peptide fragment VLSIVPSIDTGVC^58^STQTR from H_2_O_2_-treated cells, in which the cysteine residue was reduced (^13^C carboxymethylated) and oxidized (^12^C carboxymethylated), respectively. (G and H) MS/MS spectra of a triply charged fragment ion at *m/z* 487.5490 and 486.8803 for MH_2_^3+^, corresponding to the peptide FWASWC^79^GPC^82^KR of Trx from H_2_O_2_-untreated cells, in which the Cys82 residue was reduced (^13^C carboxymethylated) and oxidized (^12^C carboxymethylated), respectively. (I) MS/MS spectrum of a doubly charged fragment ion at *m/z* 729.8172 for MH_2_^2+^, corresponding to the peptide FWASWC^79^GPC^82^KR of Trx from H_2_O_2_-treated cells, in which the Cys82 residue was oxidized (^12^C carboxymethylated). (Insets) Fragments of the relevant peptide sequences matching the observed fragmentation ions. Download FIG S1, TIF file, 2.0 MB. Copyright © 2020 Tong et al. 2020 Tong et al. This content is distributed under the terms of the Creative Commons Attribution 4.0 International license.

LC-MS/MS identified 964 proteins in the samples not treated with H_2_O_2_ and 1,141 proteins in those pulsed with 40 μM H_2_O_2_; 923 were consistently detected in both samples. Among those, 93 cysteine-containing proteins were detected in H_2_O_2_-untreated cells and 132 were detected in 40 μM H_2_O_2_-treated cells (Data Set S1A to D). Proteins with reversible (S-S or SOH) or irreversible thiol oxidation (SO_2_H or SO_3_H) were identified by comparison with those in H_2_O_2_-untreated cells. The S-S oxidation ratio (in percent) in each sample was calculated by dividing the intensity of the disulfide-linked peptides by the sum of the peptides and considering a cutoff value of a ≥1.5-fold oxidation ratio in H_2_O_2_-treated cells over that in untreated cells to be significant (29). Proteins identified as SOH or SO_2_H or SO_3_H oxidations were those found only in H_2_O_2_-treated samples or with a ≥1.5-fold elevated peptide intensity compared to that for the control. In summary, 40 μM H_2_O_2_ treatment resulted in thiol group oxidation in 57 cysteine-containing proteins (Data Set S1E). Among these, 25 proteins containing 32 cysteine residues were oxidized into disulfide linkages (S-S), with a >50-fold increased disulfide ratio in 21 proteins; 3 proteins were reversibly oxidized as SOH; and the remaining 29 proteins were irreversibly oxidized as SO_2_H or SO_3_H. Thus, these 57 proteins are assumed to be involved in self-protection from H_2_O_2_ challenge.

As S. oligofermentans cells statically grown in the 40-ml culture survived the 20 mM H_2_O_2_ challenge (Table 1), we identified the cysteine-oxidized proteins in this volume of culture. A total of 1,093 proteins were identified by LC-MS/MS analysis, including 108 cysteine-containing proteins (Data Set S2A and B). Calculations indicated that 35 proteins were oxidized, with 26 cysteine residues in 23 proteins being reversibly oxidized into disulfide linkages (S-S) and the remaining 12 proteins being irreversibly oxidized as SO_2_H or SO_3_H (Data Set S2E). However, in cells cultured in 10-ml cultures that accumulated larger amounts of H_2_O_2_, 66 of the 164 cysteine-containing proteins were oxidized, with 33 being oxidized as S-S and 33 being oxidized as SO_2_H or SO_3_H (Data Set S2C to E). Thirty-one proteins that were specifically oxidized in 10- ml cultures belonged to organic acid and organic nitrogen metabolic processes (Data Set S2F), accounting for the growth retardation of S. oligofermentans under oxidative stress.

10.1128/mSystems.00006-20.9 DATA SET 1 (A) Redox proteomics-identified proteins in the S. oligofermentans wild-type strain without 40 μM H_2_O_2_ treatment. (B) Redox proteomics-identified proteins in the S. oligofermentans wild-type strain pulsed with 40 μM H_2_O_2_. (C) Redox proteomics-identified cysteine-containing proteins in the S. oligofermentans wild-type strain without 40 μM H_2_O_2_ treatment. (D) Redox proteomics-identified cysteine-containing proteins in the S. oligofermentans wild-type strain pulsed with 40 μM H_2_O_2_. (E) Reversible or irreversible oxidized cysteine-containing proteins upon H_2_O_2_ treatment in the wild-type strain. +, a peptide with an SOH or SO_2_H/SO_3_H modification was identified in the tested sample; ^a^, the thiol/disulfide oxidized ratio was calculated by dividing the intensity of the oxidized disulfide-linked peptide (^12^C carboxymethylated) over the sum of the disulfide-oxidized (^12^C-carboxymethylated) and -reduced (^13^C-carboxymethylated) peptides in the corresponding sample. (F) Overrepresented biological processes of the cysteine-oxidized proteins identified in the S. oligofermentans wild-type strain upon 40 μM H_2_O_2_ treatment Download Data Set S1, XLSX file, 0.3 MB. Copyright © 2020 Tong et al. 2020 Tong et al. This content is distributed under the terms of the Creative Commons Attribution 4.0 International license.

10.1128/mSystems.00006-20.10 DATA SET S2 (A) Redox proteomics-identified proteins in the S. oligofermentans wild-type strain grown in 40-ml cultures. (B) Redox proteomics-identified cysteine-containing proteins in the S. oligofermentans wild-type strain grown in 40-ml cultures. (C) Redox proteomics-identified proteins in the S. oligofermentans wild-type strain grown in 10-ml cultures. (D) Redox proteomics-identified cysteine-containing proteins in the S. oligofermentans wild-type strain grown in 10-ml cultures. (E) Reversible or irreversible oxidized cysteine-containing proteins identified in the S. oligofermentans wild-type strain grown in different volumes of culture medium. *, ND, the peptide fragment was not detected in the tested sample; +, the peptide with the SOH or SO_2_H/SO_3_H modification was identified in the tested sample; ^a^, the thiol/disulfide oxidized ratio was calculated by dividing the intensity of oxidized disulfide-linked peptide (^12^C carboxymethylated) by the sum of the disulfide-oxidized (^12^C-carboxymethylated) and -reduced (^13^C-carboxymethylated) peptide in the corresponding sample. (F) 1, overrepresented biological processes of the cysteine-oxidized proteins identified in the S. oligofermentans wild-type strain grown in 40-ml cultures; 2, overrepresented biological processes of the cysteine-oxidized proteins identified in the S. oligofermentans wild-type strain grown in 10-ml cultures. Download Data Set S2, XLSX file, 0.3 MB. Copyright © 2020 Tong et al. 2020 Tong et al. This content is distributed under the terms of the Creative Commons Attribution 4.0 International license.

To link the biological functions of the H_2_O_2_-sensitive proteins, Gene Ontology (GO) analysis was performed by use of the PANTHER bioinformatics platform (http://www.pantherdb.org/) (35). Figure 2B shows that the proteins oxidized by endogenous H_2_O_2_ (in 40-ml aerobic cultures) and exogenously provided H_2_O_2_ (for 40 μM H_2_O_2_-pulsed anaerobic cells) were categorized into similar biological processes, with approximately 61% and 51% of the proteins, respectively, being involved in metabolic processes and 26% and 38% of the proteins, respectively, being involved in cellular processes. Remarkably, almost all the proteins in the glycolysis and nucleotide salvage pathways were oxidized to form disulfide linkages (Table 2; Fig. 2C; Data Sets S1F and S2F). As expected, the antioxidative thiol-reducing proteins thiol peroxidase (Tpx) and thioredoxin (Trx) were 36.5% to 100% oxidized. It is worth noting that the metalloregulator MntR was markedly oxidized at the thiol group of cysteines (Table 2; Data Sets S1E and S2E). In a preliminary redox proteomic experiment, 40 μM H_2_O_2_ treatment also resulted in the oxidations of Cys139 and Cys142 of the peroxide-responsive repressor PerR (Fig. S1C). The consistently identified redox-sensitive proteins in the 40-ml cultures and 40 μM H_2_O_2_-pulsed cells (Table 2) either might be involved in self-protection or might simply be hypersensitive to oxidative damage.

**TABLE 2:** Redox proteomics identified the cysteine residues and proteins of S. oligofermentans oxidized by both an exogenous 40 μM H_2_O_2_ pulse and endogenous H_2_O_2_ produced in 40-ml static cultures

No.	Accession no. in KEGG database	Protein description	Peptide sequence	Modified cysteine	Thiol/disulfide oxidized ratio (%)^*a*^		
					40-ml culture	Nonpulsed culture	40 μM H_2_O_2_-pulsed culture
1	I872_01020	Metal-dependent transcriptional regulator	CIYEIGTR	C11	100	0	100*
2	I872_09155	Glyceraldehyde-3-phosphate dehydrogenase	TIVFNTNHDVLDGTETVISGASCTTNCLAPMAK	C151, C155	C151, 24.7; C155, 100	0	C151, SO_3_H; C155, 100*
3	I872_08015	Fructose-bisphosphate aldolase	VNVNTECQIAFANATR	C235	100	0.5 ± 0.5	100*
4	I872_06890	Pyruvate kinase	AICEETGNGHVQLFAK	C235	100	0	57.4 ± 24.9*
5	I872_00255	Aldehyde-alcohol dehydrogenase	IAEPVGVVCGITPTTNPTSTAIFK	C120	100	0	100*
6	I872_10355	6-Phospho-beta-glucosidase	NVETCLAQPVLLR	C318	100	0	100*
7	I872_00060	Hypoxanthine phosphoribosyltransferase	NLCNLFK	C112	100	0	100*
8	I872_01620	Protein translocase subunit SecA	ELGGLCVIGTER	C507	100	10 ± 10	98 ± 2*
9	I872_09640	Probable thiol peroxidase	VLSIVPSIDTGVCSTQTR	C58	100	0	36.5 ± 3.5*
10	I872_03205	Thioredoxin family protein	FWASWCGPCK^*b*^	C82	10	80	100
11	I872_00600	50S ribosomal protein L36	VMVICPANPK	C27	100	24 ± 6	100*

a The thiol/disulfide oxidized ratio was calculated by dividing the intensity of oxidized disulfide-linked peptide by the sum of the intensities of the oxidized and reduced peptides in the corresponding sample. *, a cutoff of a ≥1.5-fold oxidation ratio in H_2_O_2_-treated cells over that in untreated cells was considered significant. Experiments were repeated twice, and the results are the averages ± SD from two independent experiments.

b This peptide fragment was detected in only one independent experiment.

### Redox Western blotting validates the oxidation of cysteine-containing proteins by a low H_2_O_2_ concentration, notably, the cysteine oxidation of PerR.

**FIG 3:**
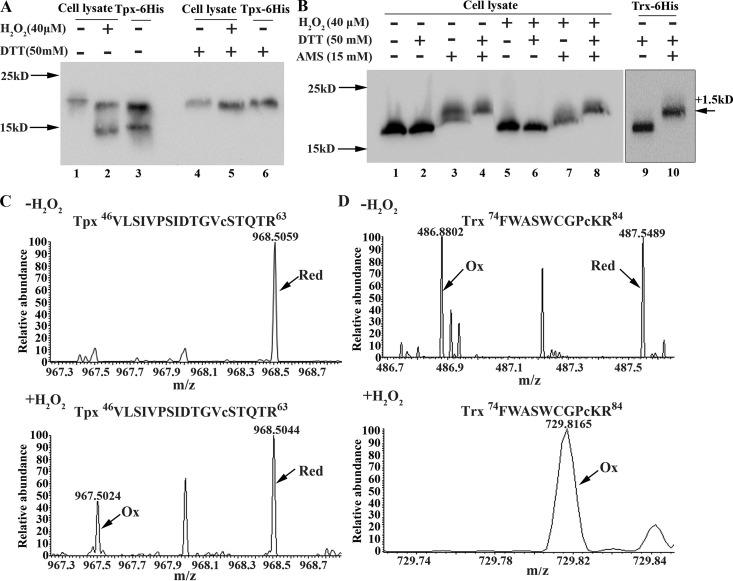
Verification of the cysteine oxidation of thiol peroxidase (Tpx) and thioredoxin (Trx) in 40 μM H_2_O_2_-treated anaerobic cultures. (A) A 6×His tag was fused to the C terminus of the *tpx* gene (KEGG accession number I872_09640) to construct the S. oligofermentans Tpx-6×His strain. Mid-exponential-phase anaerobically grown Tpx-6×His cells were treated with or without 40 μM H_2_O_2_ for 20 min, collected inside an anaerobic glovebox, and then lysed in RIPA buffer containing the free thiol protectant NEM. The cell lysate of each sample was divided into two aliquots; one was left untreated (lanes 1 and 2), and the other was reduced with 50 mM DTT for 1 h (lanes 4 and 5). Redox Western blotting was carried out using an 18% SDS-PAGE gel to detect the Tpx-6×His protein using an anti-His tag antibody. Recombinant Tpx-6×His protein, which was partially oxidized and which formed an intramolecular disulfide linkage during purification, was treated with or without 50 mM DTT (lanes 3 and 6) and used as a reduced and an oxidized molecular control, respectively. (B) Using the same approach described in the legend to panel A, a disulfide linkage upon 40 μM H_2_O_2_ oxidation was identified for thioredoxin (KEGG accession number I872_03205) in the Trx-6×His strain (lanes 1 and 2 versus lanes 5 and 6). In addition, 15 mM 4-acetamido-4′-maleimidylstilbene-2,2′-disulfonic acid (AMS), the free thiol-chelating reagent, was used to detect the nondisulfide oxidation of the thiol groups (lanes 3 and 4 and lanes 7 and 8). Cell lysates from the H_2_O_2_-untreated strain (lanes 3 and 4) and the H_2_O_2_-treated Trx-6×His strain (lanes 7 and 8) were reduced with or without 50 mM DTT. The recombinant Trx-6×His protein was first reduced by 50 mM DTT, and then one aliquot was alkylated with AMS and another was left untreated; these were used as reduced and thiol AMS-bound Trx-6×His protein controls, respectively (lanes 9 and 10). Molecular weight markers are shown at the left, and the increased molecular weight of the protein due to bound AMS molecules (500 Da each) is shown at the right. (C and D) Redox proteomics identified the reduced (Red) and oxidized (Ox) peptide fragments of Tpx VLSIVPSIDTGVC^58^STQTR (C) and Trx FWASWCGPC^82^KR (D) in H_2_O_2_-untreated (top) and H_2_O_2_-treated S. oligofermentans cells (bottom). The relative abundances of the oxidized and reduced peptide fragments are shown.

To verify the redox proteomics-identified cysteine residues sensitive to low H_2_O_2_ concentrations, Tpx and Trx, the well-known antioxidative proteins, were chosen to examine cysteine oxidation by 40 μM H_2_O_2_ in Tpx-6×His and Trx-6×His strains, which carried a 6×His tag fusion at the C terminus of the Tpx and Trx proteins, respectively. Redox Western blotting was performed as described in Materials and Methods. As shown in Fig. 3A, in comparison with the migration of Tpx from H_2_O_2_-untreated Tpx-6×His cells (lane 1), a similar faster-migrating band was also detected for both Tpx from H_2_O_2_-treated cells (lane 2) and the recombinant Tpx-6×His protein (lane 3), which could be partially oxidized during purification, while upon dithiothreitol (DTT) reduction, the faster-migrating Tpx band from H_2_O_2_-treated cells and the Tpx-6×His protein disappeared (lanes 4, 5, and 6). This indicates that H_2_O_2_ oxidation results in an intramolecular disulfide linkage in Tpx. Consistently, LC-MS/MS identified the thiol oxidation of Cys58 (Fig. 3C; Fig. S1E and F), and H_2_O_2_ treatment caused approximately 36% of the Tpx protein to be oxidized (Fig. 3C; Table 2; Data Set S1E). Although redox proteomics identified Trx Cys82 to be complete oxidized (Fig. 3D; Fig. S1I), redox Western blotting did not detect a differential migration of the Trx protein upon H_2_O_2_ oxidation or DTT reduction (Fig. 3B, lanes 1 and 2 versus lanes 5 and 6), while addition of 4-acetamido-4′-maleimidylstilbene-2,2′-disulfonic acid (AMS), a free thiol-reactive reagent, generated different upshifted Trx bands by higher upshifting in DTT-reduced cells than in H_2_O_2_-treated cells (Fig. 3B, lanes 4 and 8 versus lanes 3 and 7). By reference to an apparent molecular weight increase of 500 Da per AMS molecule (36) and the migration of three AMS-bound recombinant Trx-6×His proteins (Fig. 3B, lane 10), the differential protein migration in H_2_O_2_-treated and DTT-reduced cells suggests that a reversible thiol group oxidation (–SOH) in one cysteine of Trx is formed by H_2_O_2_ treatment. Therefore, redox Western blotting confirmed the oxidation of cysteine residues in cells treated with a low concentration of H_2_O_2_.

**FIG 4:**
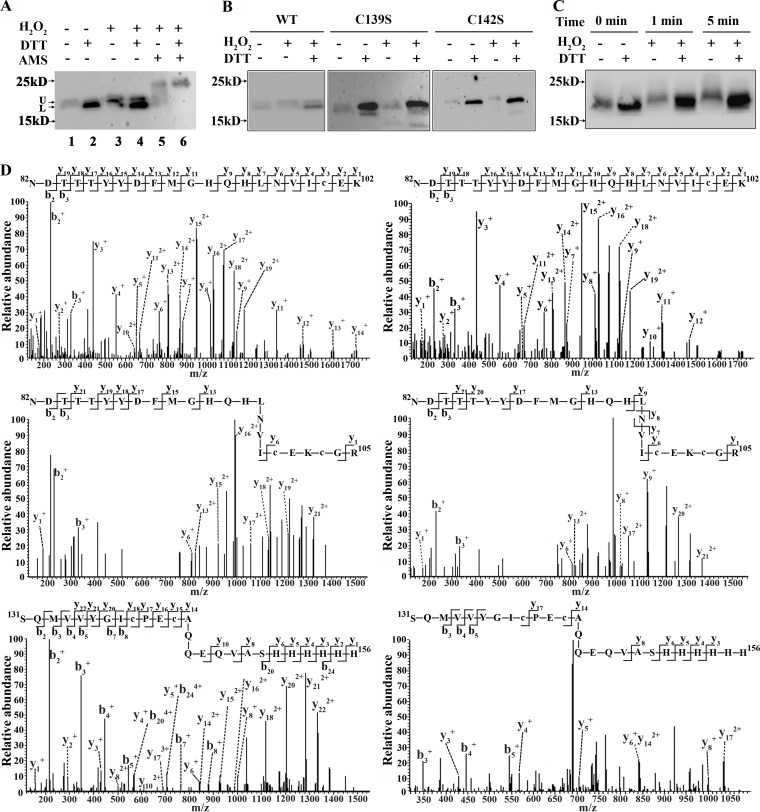
Assay of PerR cysteine oxidation in 40 μM H_2_O_2_-treated cells. (A) A 6×His tag was fused to the C terminus of *perR* (KEGG accession number I872_05555) to construct the S. oligofermentans PerR-6×His strain. Using the same approach described in the legends to Fig. 3A and B, redox Western blotting detected cysteine oxidation in the 6×His-tagged PerR protein using the anti-His tag antibody. U and L at the gel left indicate upper and lower protein bands, respectively. (B) A serine substitution of either Cys139 or Cys142 was constructed on a shuttle plasmid (pDL278-*perR*-6×His), and the plasmid was transformed into the *perR* deletion strain to construct the *perR*::pDL278-*perR*C139S-6×His (C139S) and *perR*::pDL278-*perR*C142S-6×His (C142S) strains. The *perR* deletion mutant harboring pDL278-*perR*-6×His (WT) was included as a control. The three strains were anaerobically cultured and then treated with 40 μM H_2_O_2_. Redox Western blotting, as described in the legend to Fig. 3A, was carried out to detect the oxidation of the PerR mutants. (C) The PerR-6×His strain was anaerobically cultured, and cells were treated with 40 μM H_2_O_2_ for 1 min and 5 min. Using the methods described in the legend to Fig. 3A, the cysteine oxidation of PerR was detected by redox Western blotting. (D) The 6×His-tagged PerR protein was immunoprecipitated from the statically grown PerR-6×His strain as described in Materials and Methods and then resolved on an 18% nonreducing SDS-PAGE gel. The protein band was then subjected to differential alkylation and LC-MS/MS analysis. (Top) Representative MS/MS spectra of the triply charged peptide ions at *m/z* 863.7217 and 862.7243, corresponding to reduced (left) and oxidized (right) NDTTTYYDFMGHQHLNVIC^100^EK peptide fragments, respectively. (Middle) Representative MS/MS spectra of triply charged peptide ions at *m/z* 989.1003 and 992.4445, corresponding to both Cys100 and Cys103 reduced (left) and oxidized (right) NDTTTYYDFMGHQHLNVIC^100^EKC^103^GR peptide fragments, respectively. (Bottom) The MS/MS spectra represent triple- and quintuple-charged peptide ions at *m/z* 1038.1281 and 622.0834, respectively, corresponding to both Cys139 and Cys142 reduced (left) and oxidized (right) SQMVVYGIC^139^PEC^142^AQQEQVASHHHHHH peptide fragments. The reduced and oxidized cysteine residues were ^13^C carboxymethylated and ^12^C carbamidomethylated, respectively.

Previously, we demonstrated that the peroxide-responsive repressor PerR and the metalloregulator MntR are involved in the H_2_O_2_ resistance of S. oligofermentans (11, 25). Interestingly, redox proteomics detected the cysteine oxidation of the two proteins in 40 μM H_2_O_2_-treated cells. Recently, we have validated the increased amount of disulfide-linked MntR oligomer in 40 μM H_2_O_2_-pulsed cells (25). Here, we examined H_2_O_2_-caused cysteine oxidation in PerR. A PerR-6×His strain, which carries a 6×His tag fusion at the C terminus of PerR, was treated with or without 40 μM H_2_O_2_ and then lysed in the presence of the free thiol protectant *N*-ethylmaleimide (NEM) and 10 mM EDTA, which chelates Fe^2+^ and so avoids Fenton chemistry-mediated PerR oxidation, as demonstrated in B. subtilis PerR (20). Redox Western blotting detected two bands in the H_2_O_2_-untreated PerR-6×His culture, whereas the upper band appeared mainly in the H_2_O_2_-treated culture and the lower one appeared exclusively in DTT-treated cell lysates (Fig. 4A, lanes 1 to 4, and Fig. 4B). This is reminiscent of the findings for B. subtilis PerR, which migrated more slowly when the structure maintaining Zn^2+^ was lost due to the oxidation of cysteine residues (20). Therefore, AMS was employed to examine the cysteine redox status of PerR from H_2_O_2_-treated PerR-6×His cells. Figure 4A shows that AMS addition increased the apparent molecular weight of PerR from DTT-reduced cell lysates (upshifted at approximately 0.5 cm, lane 6 versus lane 4) compared to that of PerR from the non-DTT-reduced ones (upshifted at approximately 0.3 cm, lane 5 versus lane 3). This indicates that some but not all of the four Cys residues of the streptococcal PerR are oxidized by pulsing with a low H_2_O_2_ concentration.

Redox Western blotting indicated that PerR was also oxidized in statically grown cells (Fig. S2A). To further verify the endogenous cysteine oxidation resulting from H_2_O_2_, 6×His-tagged PerR protein immunoprecipitated from statically grown cells was subjected to differential alkylation and LC-MS/MS analysis (Fig. S2B). Figure 4D displays the representative MS/MS spectra of the peptide fragments carrying four cysteine residues. By counting the peptide fragments carrying oxidative and reductive cysteine residues, we calculated the oxidation ratios of the four cysteine residues to be 76% (Cys100), 50% (Cys103), 83% (Cys139), and 82% (Cys142) (Table S1), whereas His40 and His95, whose oxidations inactivate B. subtilis PerR (20), were oxidized approximately 28% and 53%, respectively, in S. oligofermentans PerR (Table S1). Collectively, both MS/MS identification and redox Western blotting determined that the Zn^2+^-coordinated cysteine residues of S. oligofermentans PerR are hypersensitive to H_2_O_2_ oxidation.

10.1128/mSystems.00006-20.2 FIG S2 Redox Western blotting detects *in vivo* PerR oxidation (A), and immunoprecipitation detects oxidized PerR (B). (A) Redox Western blotting determined that the PerR proteins were reversibly oxidized in aerobically grown cells. Mid-exponential-phase S. oligofermentans PerR-6×His cells were sonicated in RIPA buffer containing the free thiol protectant NEM and 10 mM EDTA. Cell lysates were divided into two aliquots, in which one was treated with 50 mM DTT for 1 h and one was left untreated. Redox Western blotting was performed to assay the oxidative status of the PerR-6×His protein using anti-6×His antibody, and the molecular weight marker is labeled at the left. (B) Immunoprecipitation of the oxidized cellular PerR. One liter of mid-exponential-phase cells of the statically grown PerR-6×His strain was collected, washed twice with PBS, and resuspended in 10 ml lysis buffer. The 6×His-tagged PerR protein was pulled down using anti-His tag monoclonal antibody-magnetic beads as described in Materials and Methods. Proteins in the elution (IP elution) were separated on an 18% nonreducing SDS-PAGE gel and stained with Coomassie brilliant blue. The molecular mass marker is labeled at the left, and the black arrow indicates the target 6×His-tagged PerR protein with a predicted molecular weight of 17.8 kDa. Download FIG S2, TIF file, 0.2 MB. Copyright © 2020 Tong et al. 2020 Tong et al. This content is distributed under the terms of the Creative Commons Attribution 4.0 International license.

10.1128/mSystems.00006-20.6 TABLE S1 Calculation of the oxidation ratio of cysteine or histidine residues in 6×His-tagged PerR that was immunoprecipitated from statically grown S. oligofermentans. ^a^, the numbers of LC-MS/MS-identified peptide spectral matches (PSMs) of the fragments containing targeting amino acid residues were counted, and the oxidation ratios of the respective residues were calculated by dividing the number of oxidized PSMs by the total number of PSMs. Download Table S1, DOCX file, 0.02 MB. Copyright © 2020 Tong et al. 2020 Tong et al. This content is distributed under the terms of the Creative Commons Attribution 4.0 International license.

### H_2_O_2_ oxidation of the cysteine residues abolishes PerR binding to DNA due to Zn^2+^ loss.

To further determine whether H_2_O_2_ oxidation occurs at the cysteine residues of the streptococcal PerR *in vivo*, Cys139 and Cys142 were replaced by serine on the shuttle plasmid pDL278-*perR*-6×His. Wild-type *perR* and cysteine-mutated *perR* were each ectopically expressed in a *perR* deletion strain, and the resultant complementary strains were treated with or without 40 μM H_2_O_2_. Redox Western blotting showed that, different from the findings for wild-type PerR, cysteine-mutated PerR retained the same migration in all samples regardless of 40 μM H_2_O_2_ oxidation or DTT reduction (Fig. 4B). This result demonstrates that H_2_O_2_ oxidizes Cys139 and Cys142 of the streptococcal PerR.

It is worth noting that even if cells were collected inside an anaerobic glove box and lysed in the presence of NEM, EDTA, and catalase, part of the PerR protein was still oxidized (Fig. 4A, lane 1), suggesting the PerR cysteine residues are hypersensitive to oxidants. This was further confirmed by redox Western blotting, which detected oxidized PerR protein from the cells pulsed by 40 μM H_2_O_2_ for only 1 min (Fig. 4C). Noticeably, invariable lower Western blotting signals were detected for PerR from H_2_O_2_-treated cells than for PerR from DTT-reduced cells, and this was determined to be because DTT increased the anti-His tag antibody signal (Fig. S3).

10.1128/mSystems.00006-20.3 FIG S3 DTT reduction increases the hybridization signal of the anti-His tag antibody. Overnight cultures of *perR*::pDL278-*perR*C142S-6×His strain were diluted 1:30 into fresh BHI broth and grown statically. The mid-exponential-phase cells were collected, washed twice with PBS, and sonicated in RIPA buffer containing the free thiol protectant NEM and 10 mM EDTA. The cell lysate was divided into seven aliquots; six of these were treated with gradient concentrations of DTT (lanes 2 to 7) for 1 h, and one was not treated with DTT. Redox Western blotting on 18% SDS-PAGE gels was performed to assay for the PerR-C142S protein using the anti-His tag antibody. The black arrow indicates the PerR-C142S hybridization band; the gray arrow shows the molecular weight marker; *, nonspecific hybridization band. Download FIG S3, TIF file, 0.1 MB. Copyright © 2020 Tong et al. 2020 Tong et al. This content is distributed under the terms of the Creative Commons Attribution 4.0 International license.

**FIG 5:**
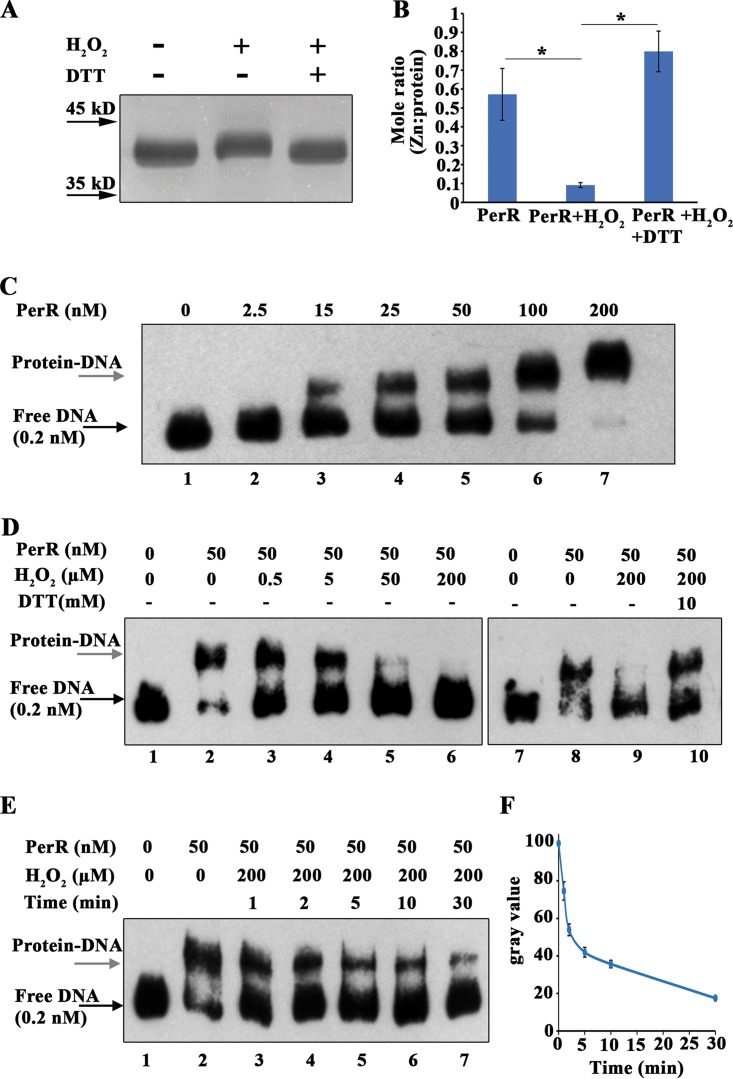
Determination of zinc ion loss and inactivation of the PerR protein caused by H_2_O_2_ oxidation. (A and B) The recombinant PerR-GST protein was purified in PBS buffer containing 10 mM EDTA and 3 mM GSH, as described in Materials and Methods. Purified PerR-GST was divided into three aliquots: one was not treated, and the remaining two were treated for 30 min with 5 mM H_2_O_2_, with one of these two aliquots subsequently being subjected to 1 h of reduction with 50 mM DTT. Aliquots of the three protein samples were resolved on nonreducing 18% SDS-PAGE gels, and the remaining samples were ultrafiltered and, finally, resuspended in 650 μl PBS buffer. The protein concentrations of PerR-GST were determined using a BCA protein assay kit, and the zinc concentration in the protein was measured using ICP-MS. The molar ratios of zinc ion to the PerR-GST monomer were calculated. The averages ± SD from three independent experiments are shown. *, a result significantly different from that for H_2_O_2_-treated PerR-GST protein, as verified by one-way analysis of variance followed by Tukey’s *post hoc* test (*P < *0.05). (C) The PerR-GST protein was digested with 100 U thrombin to remove the GST tag and then eluted into a buffer containing 10 mM EDTA and 1 mM DTT. Next, 1 μM PerR protein was preincubated with 1 mM MnCl_2_, and then gradient concentrations of the recombinant PerR:Zn,Mn protein (0 to 200 nM) were tested for binding to the 5′-biotin-labeled *dpr* promoter fragment, as described in Materials and Methods. (D) One micromole of PerR protein was incubated with 1 mM MnCl_2_ and then treated with increasing concentrations of H_2_O_2_ at 30°C for 30 min. After incubation, 150-U/ml catalase was added to decompose the residual H_2_O_2_, and 50 nM PerR protein was used for EMSA to test the affinity of binding to the *dpr* promoter (lanes 3 to 6). To observe whether the H_2_O_2_ oxidation-diminished PerR binding could be restored, 10 mM DTT was added to the 200 μM H_2_O_2_-treated PerR and the mixture was incubated at 37°C for 1 h (lane 10). E. One micromole of the PerR protein was preincubated with 1 mM MnCl_2_ and then treated with 200 μM H_2_O_2_ at 30°C for different times (lanes 3 to 7). Fifty nM PerR protein was used in the EMSA. Black and gray arrows point to the free DNA probe and protein-DNA complex, respectively. (F) Band densities of the protein-DNA complex in lanes 2 to 7 of panel E were evaluated using ImageJ software, with the density in lane 2 being set as 100%. The density percentages in lanes 3 to 7 were calculated by dividing the band density of the respective lane by that of lane 2. All the experiments were repeated three times, and the averages ± SD from three independent experiments are shown.

By reference to the B. subtilis PerR and other Cys_4_Zn proteins, such as Hsp33 and RsrA (37, 38), oxidation of Zn^2+^-coordinated cysteine residues would cause Zn^2+^ loss and, therefore, slower protein migration because of the conformational changes. We subsequently verified whether H_2_O_2_ oxidation causes Zn^2+^ loss from PerR. Overexpressed glutathione *S*-transferase (GST)-tagged PerR protein (GST-PerR) was treated or not treated with 5 mM H_2_O_2_ and subsequently reduced or not reduced with 50 mM DTT. Nonreducing SDS-PAGE analysis did reveal a slower-migrating band for the 5 mM H_2_O_2_-treated protein than for the DTT-treated protein (Fig. 5A). Inductively coupled plasma mass spectrometry (ICP-MS) also determined 0.09 mol of Zn^2+^ per mol of H_2_O_2_-treated PerR and 0.79 mol of Zn^2+^ per mol of DTT-reduced PerR (Fig. 5B), confirming that H_2_O_2_ oxidation causes Zn^2+^ loss from PerR.

Next, we determined whether Zn^2+^ loss affects DNA binding by PerR. Fifty nanomoles of PerR:Zn,Mn was used for a electrophoretic mobility shift assay (EMSA), based on a calculated *K_d_* (dissociation constant) value of approximate 50 nM for binding (Fig. 5C). Figure 5D shows that 50 μM H_2_O_2_ treatment for 30 min diminished PerR’s binding to the *dpr* promoter (lane 5) and that 200 μM H_2_O_2_ treatment completely abolished the binding (lane 6); meanwhile, 10 mM DTT reduction recovered the binding (lane 9 versus lane 10), indicating that PerR is reversibly inactivated by H_2_O_2_ oxidation at cysteine residues. Moreover, 5 min of treatment with 200 μM H_2_O_2_ inactivated approximately 59% of the PerR protein (Fig. 5E and F). It is worth noting that the EMSA buffer was treated with Chelex 100 to chelate metal ions that might trigger the Fenton reaction. Noticeably, LC-MS/MS did not detect increased histidine residue oxidation in the H_2_O_2_-treated PerR:Zn,Mn protein (Table S2), while histidine oxidations that occurred before H_2_O_2_ treatment might have been generated during the *in vitro* purification. In conclusion, H_2_O_2_ oxidizes cysteine residues but not histidine residues and inactivates PerR.

10.1128/mSystems.00006-20.7 TABLE S2 Calculation of histidine oxidation in the recombinant PerR:Zn,Mn protein resulting from H_2_O_2_. ^a^, the numbers of LC-MS/MS-identified peptide spectral matches (PSMs) of the fragments containing histidine residues were counted, and the oxidation ratios of the histidine residues were calculated by dividing the number of oxidized PSMs by the total number of PSMs. Download Table S2, DOCX file, 0.02 MB. Copyright © 2020 Tong et al. 2020 Tong et al. This content is distributed under the terms of the Creative Commons Attribution 4.0 International license.

### PerR and MntR regulate the cellular redox system and metal homeostasis.

Trx and Tpx are involved in cellular redox homeostasis and belong to the S. aureus and Clostridium acetobutylicum PerR regulons (39, 40). Additionally, Dpr, a non-heme iron-containing ferritin, and MntABC, a manganese ABC transporter, are known to play important roles in maintaining cellular metal homeostasis and are under the control of S. oligofermentans PerR and MntR (11, 25). To determine whether the four genes mentioned above plus *mntR* belong to the PerR or MntR regulons, we performed quantitative PCR (qPCR) to quantify the expression of these genes in 40 μM H_2_O_2_-pulsed and nonpulsed anaerobically grown wild-type strain and *perR* deletion, *mntR* deletion, and *perR mntR* double deletion mutants. In comparison with the 40 μM H_2_O_2_-induced 3- to 5.8-fold higher levels of expression of *tpx*, *dpr*, *mntA*, and *mntR* in the wild-type strain, deletion of *mntR* abolished the H_2_O_2_ induction of *mntA*; however, the H_2_O_2_ induction of the four genes almost disappeared in the mutants either with a deletion of *perR* or with a deletion of both *perR* and *mntR* (Table 3). These demonstrate that the H_2_O_2_-induced expressions of *tpx*, *dpr*, *mntA*, and *mntR* are under the control of PerR, while *mntA* is also controlled by *mntR* in response to H_2_O_2_.

**TABLE 3:** Identification of the PerR regulon by qPCR quantification of the gene transcript copies in anaerobically grown wild-type and *perR* and *mntR* single and double deletion strains with and without 40 μM H_2_O_2_ treatment

Gene^*a*^	Transcript copy no./100 16S rRNA copies^*b*^								
	Wild-type strain			Δ*perR* mutant		Δ*mntR* mutant		Δ*perR* Δ*mntR* mutant	
	Without H_2_O_2_	With H_2_O_2_	With H_2_O_2_ + high^*c*^	Without H_2_O_2_	With H_2_O_2_	Without H_2_O_2_	With H_2_O_2_	Without H_2_O_2_	With H_2_O_2_
*mntR*	0.76 ± 0.05	2.35 ± 0.21*	2.16 ± 0.31*	0.77 ± 0.12	0.83 ± 0.02	ND	ND	ND	ND
*mntA*	5.52 ± 1.50	18.04 ± 3.75*	20.75 ± 1.20*	13.81 ± 0.93*	21.70 ± 3.94*	18.70 ± 1.59*	19.70 ± 0.36*	18.94 ± 2.02*	22.47 ± 3.53*
*dpr*	6.26 ± 2.18	34.92 ± 6.40*	29.54 ± 4.74*	21.40 ± 2.80*	27.91 ± 0.16*	7.89 ± 0.76	23.62 ± 2.47*#	25.01 ± 0.26*	30.77 ± 6.82*
*tpx*	0.56 ± 0.11	3.22 ± 0.17*	3.10 ± 0.23*	1.30 ± 0.19*	1.45 ± 0.24*	0.66 ± 0.02	1.78 ± 0.10*#	1.60 ± 0.08*	1.71 ± 0.39*
*trx*	0.73 ± 0.17	1.35 ± 0.35	0.75 ± 0.34	0.96 ± 0.12	1.36 ± 0.18	0.82 ± 0.19	0.79 ± 0.09	0.62 ± 0.02	0.80 ± 0.16

a *mntR*, Mn-dependent transcriptional regulator (KEGG accession number I872_01020); *mntA*, manganese transport system substrate-binding protein (KEGG accession number I872_09645); *dpr*, non-heme iron-containing ferritin (KEGG accession number I872_07415); *tpx*, thiol peroxidase (KEGG accession number I872_09640); *trx*, thioredoxin (KEGG accession number I872_03205).

b The experiments were repeated three times with triplicate batch cultures each time. The results are the averages ± SD from three independent experiments. The data were significantly different from those obtained for the wild-type strain without 40 μM H_2_O_2_ treatment (*) and from those obtained with the same strain not treated with H_2_O_2_ (#), as verified by one-way analysis of variance followed by Tukey’s *post hoc* test (*P *< 0.05). ND, not determined.

c qPCR was implemented with the wild-type strain prepulsed with 40 μM H_2_O_2_ and then further challenged by 10 mM H_2_O_2_.

Notably, even in the absence of H_2_O_2_, 2.5- to 4.0-fold higher levels of expression of *tpx*, *dpr*, and *mntA* were detected in the Δ*perR* and Δ*perR* Δ*mntR* mutants than in the wild-type strain, suggesting that PerR may directly regulate the *tpx*, *dpr*, and *mntA* genes. However, the conserved PerR binding sequence (TTAATTAGAAGCATTATAATTAA) was found only in the *dpr* promoter region; consistently, EMSA indicated PerR binding to the *dpr* promoter (Fig. 5C) but not to the promoters of *mntABC*, *tpx*, and *mntR* (Fig. S4). Our previous work found that MntR bound to the *mntABC* promoter (25), indicating the direct regulation of *mntA* by MntR. Thus, PerR directly regulates *dpr* but indirectly regulates *mntA*, *tpx*, and *mntR* via unknown mechanisms. Of note, similar expression levels of the PerR-regulated genes were detected in cells pulsed only with 40 μM H_2_O_2_ and cells that were pulsed and then further challenged by 10 mM H_2_O_2_ (Table 3), indicating that these genes are under the control of PerR, which is already inactivated by as little as 40 μM H_2_O_2_.

10.1128/mSystems.00006-20.4 FIG S4 EMSA of PerR binding to the *mntABC*, *tpx*, and *mntR* promoters. The PerR-GST protein was digested with 100 U thrombin to remove the GST tag and then eluted into a buffer containing 10 mM EDTA and 1 mM DTT. Next, 1 μM PerR protein was preincubated with 1 mM MnCl_2_, and then gradient concentrations of the recombinant PerR:Zn,Mn protein (0 to 200 nM) were used for binding to 5′-biotin-labeled *mntABC* (KEGG accession numbers I872_09645, I872_09650, and I872_09655), *tpx* (KEGG accession number I872_09640), and *mntR* (KEGG accession number I872_01020) promoters, as described in Materials and Methods. Black arrows point to the free DNA probes (0.2 nM for each). Download FIG S4, TIF file, 0.6 MB. Copyright © 2020 Tong et al. 2020 Tong et al. This content is distributed under the terms of the Creative Commons Attribution 4.0 International license.

### PerR, MntR, and the regulated cellular redox and metal homeostatic proteins are involved in the self-protection of S. oligofermentans from H_2_O_2_ stress.

Given that both PerR and MntR contribute to the high H_2_O_2_ resistance of S. oligofermentans (11, 25), to determine their roles in self-protection against H_2_O_2_ stress, the Δ*perR* and Δ*mntR* mutants were prepulsed with or without 40 μM H_2_O_2_, and then their survival with 10 mM H_2_O_2_ challenge was determined as described above. Table 1 shows that the *mntR* deletion dramatically reduced the 40 μM H_2_O_2_-induced protection from high H_2_O_2_ challenge to 8-fold, compared to the 367-fold protection in the wild-type strain, while *perR* deletion almost completely abolished the low-H_2_O_2_-concentration-induced adaptation (Table 1). Together, the two redox regulators MntR and, in particular, PerR play important roles in the low-H_2_O_2_-concentration-induced self-protection of S. oligofermentans from a higher-concentration H_2_O_2_ stress, most likely by H_2_O_2_ inactivating the two transcriptional repressors and thereby derepressing the antioxidative systems. Notably, a 9.4-fold elevated survival rate in the presence of a higher concentration of H_2_O_2_ was observed for the Δ*perR* mutant than for the Δ*mntR* mutant (Table 1), suggesting that Dpr and redox system proteins might play the major role in protecting S. oligofermentans from challenge with a higher H_2_O_2_ concentration.

**FIG 6:**
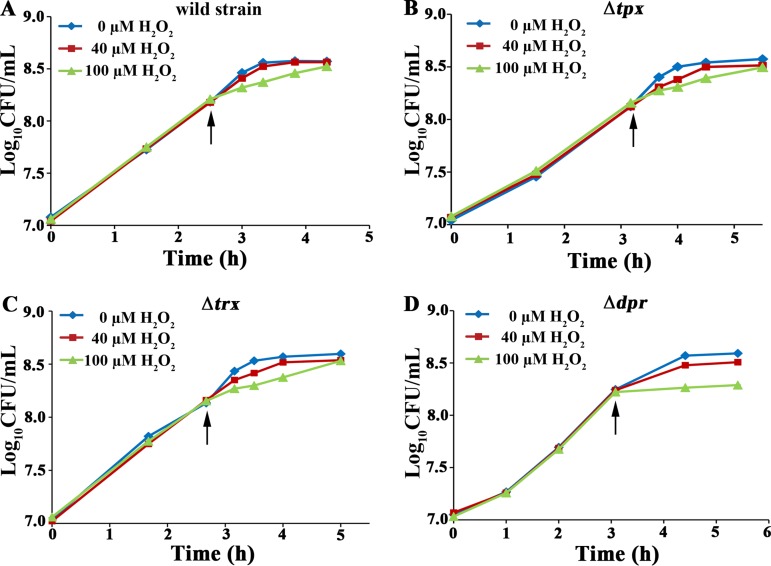
H_2_O_2_ sensitivity assay of mutants with deletions of the genes involved in cellular redox and metal homeostasis. Overnight cultures of the tested strains were diluted 1:30 into fresh BHI broth and anaerobically cultured. Triplicate cultures were used for each strain. When the OD_600_ reached approximately 0.5, one replicate was left as the H_2_O_2_-untreated control and the other two were supplemented with 40 and 100 μM H_2_O_2_, respectively. The growth profiles of the tested strains were monitored by counting the number of CFU at the indicated time points. The experiments were repeated three times, with triplicate cultures being used each time. The averages from three independent experiments are shown. Black arrows indicate the time point of H_2_O_2_ supplementation.

Next, the role of Dpr and redox system proteins in the H_2_O_2_ resistance of S. oligofermentans was determined. The *tpx*, *trx*, *dpr*, and *mntABC* genes were each deleted, and the mutants were compared with the wild-type strain for growth suppression by 40 and 100 μM H_2_O_2_. As shown in Fig. 6, 100 μM H_2_O_2_ slightly suppressed the growth of the wild-type strain, but 40 μM H_2_O_2_ already retarded the growth of the *tpx*, *trx*, and *dpr* deletion mutants, with the Δ*dpr* mutant being the most severely inhibited. The Δ*mntABC* mutant was reported to exhibit reduced resistance to 10 mM H_2_O_2_ (11), but its growth was not significantly inhibited by 100 μM H_2_O_2_ (data not shown). Collectively, the results indicate that the redox regulators PerR and MntR and their regulated cellular redox and metal homeostasis proteins are involved in the self-protection of S. oligofermentans from H_2_O_2_ stress.

## DISCUSSION

**FIG 7:**
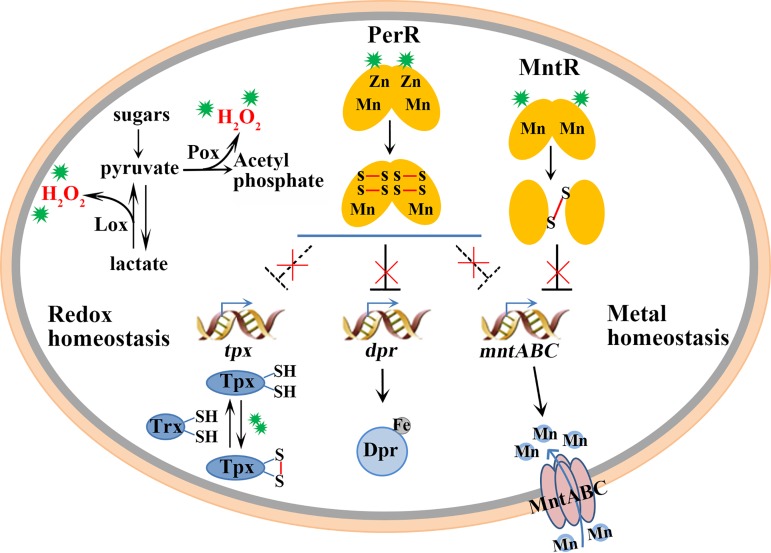
Diagram depicting the low-H_2_O_2_-concentration-induced adaptive mechanism against higher H_2_O_2_ stress in streptococci. The catalase-void streptococci use pyruvate oxidase (Pox) and lactate oxidase (Lox) to generate low levels of endogenous H_2_O_2_, which induces an adaptation to avoid attack by a higher H_2_O_2_ concentration. Redox proteomics analysis and physiological and genetic experiments identified a hierarchal H_2_O_2_-sensing and resistance network consisting of the H_2_O_2_-sensitive cysteine-containing proteins. These include redox transcriptional regulators, e.g., the peroxide response repressor PerR and the metalloregulator MntR, a repressor of the Mn^2+^ uptake regulon *mntABC*, as well as the redox homeostatic proteins, e.g., thiol peroxidase (Tpx), which catalyzes the reduction of H_2_O_2_, and thioredoxin (Trx), which specifically reduces H_2_O_2_ oxidation-generated disulfide linkages. Inactivation of PerR by trace H_2_O_2_ derepresses *tpx* and the genes encoding metal ion homeostatic proteins, like *mntR*, *dpr*, and *mntABC*, whereas oxidation inactivation of MntR derepresses *mntABC* (25). Dpr chelates free ferrous ion to avoid Fenton chemistry, whereas MntABC imports Mn^2+^ to decompose the cellular H_2_O_2_. These functional proteins help S. oligofermentans resist the stress associated with a higher H_2_O_2_ concentration. Of note, PerR directly represses the *dpr* gene and controls the *tpx*, *mntABC*, and *mntR* genes indirectly by unknown mechanisms. H_2_O_2_ is identified by the green symbols.

Although a few studies have reported that endogenous H_2_O_2_ protects streptococci from challenge with a higher H_2_O_2_ concentration (8, 22), the mechanism remains unclear. In the present study, through a combination of physiological, biochemical, genetic, and redox proteomic studies, we elucidated the mechanism underlying the low-H_2_O_2_-concentration-induced adaptation of catalase-negative streptococci to a higher H_2_O_2_ concentration. Figure 7 depicts that streptococci employ pyruvate oxidase (Pox) and lactate oxidase (Lox) to produce endogenous H_2_O_2_. Two H_2_O_2_-sensing redox regulators, the peroxide-responsive repressor PerR and the metalloregulator MntR, are inactivated by H_2_O_2_ oxidation of the cysteine residues. PerR cysteine oxidation results in Zn^2+^ loss and the subsequent derepression of *dpr*, *mntABC*, *tpx*, and *mntR*. H_2_O_2_ oxidation of MntR leads to disulfide-linked intermolecular polymers and inactivates the regulator, thus derepressing the manganese uptake regulon *mntABC* (25). In addition to *dpr* and *mntABC*, as indicated in our previous work (11), *mntR* and the thiol peroxidase-encoding gene *tpx* were identified to be the PerR regulons. Deletion of these functional genes as well as the redox circuit protein Trx increased the sensitivity of S. oligofermentans to a low H_2_O_2_ concentration, and correspondingly, deletion of either *mntR* or *perR* resulted in the streptococci becoming constitutively resistant to a higher H_2_O_2_ concentration. Thus, this work reveals a redox-regulated anti-H_2_O_2_ defense network, in which PerR has evolved to sense H_2_O_2_ by a Cys-based redox reaction in the manganese-rich cellular environments of the catalase-negative streptococci.

Cysteine residues are the most sensitive to H_2_O_2_ oxidation (41), and therefore, reversibly oxidized cysteine thiol modifications, such as SOH and the disulfide bond, usually function in the activation of redox regulatory proteins. Some redox regulators, such as the E. coli chaperone protein Hsp33 (37) and the Streptomyces coelicolor anti-sigma factor RsrA (38) and Fur-like repressor CatR (42), possess a structural Cys_4_Zn. The Zn^2+^ at the Cys_4_Zn site stabilizes the cysteine residues as thiolate, which may increase the reactivity of cysteine toward electrophilic H_2_O_2_ (43). The S. oligofermentans PerR possesses the structural Cys_4_Zn as well. Redox proteomics, redox Western blotting, and LC-MS/MS identification of the immunoprecipitated protein all demonstrated that the cysteine residues of the streptococcal PerR are oxidized by a low H_2_O_2_ concentration (Fig. 4). Oxidation of the cysteine residues causes Zn^2+^ loss and inactivates PerR (Fig. 5), thereby derepressing the antioxidative genes (Table 3). This explains the underlying mechanism of the PerR-mediated H_2_O_2_ adaptation of streptococci.

It has been reported that the B. subtilis PerR, an ortholog of the streptococcal PerR (see Fig. S5 in the supplemental material), is inactivated by histidine oxidation, whereas its Zn^2+^-coordinated cysteine residues are inert to H_2_O_2_ oxidation (20). In contrast, the streptococcal PerR is inactivated by H_2_O_2_ oxidation at the Zn^2+^-coordinated cysteine residues (Fig. 4 and 5). Subsequent structural homology modeling of the S. oligofermentans PerR was performed with the SWISS-MODEL server by automatically selecting the S. pyogenes PerR (PDB accession number 4LMY) as a template. Structural comparison with B. subtilis PerR (PDB accession number 2FE3) did show some differences between the two at the C-terminal Cys_4_Zn site (Fig. S5); specifically, Cys103 and Cys142 of the S. oligofermentans PerR are situated close to the N terminus of the S4 β-strand and at a short H6 helix, respectively, while Cys96 and Cys139 of the B. subtilis PerR are situated at the C terminus of the S3 β-strand and in the center of a long H6 helix, respectively. These differences may render the two PerRs with different H_2_O_2_ sensitivities, as a cysteine residue near the N terminus of a helix more likely possesses lower pK_a_ values (43). Cellular metal environments could be another clue to the distinct inactivation mechanisms of the two PerRs. A much higher ratio of Mn/Fe was determined in S. oligofermentans cells (1.02 ± 0.25) than in B. subtilis cells (0.05 ± 0.01), which was paralleled in this study, in accordance with the Mn-centric definition of streptococci (44–46). Especially, when grown in a medium supplemented with 2.5 μM and 100 μM Mn^2+^, 1.78 ± 0.46 and 8.04 ± 0.42 cellular Mn/Fe ratios were found in S. oligofermentans, respectively (25), indicating an active manganese uptake system in this bacterium. The higher cellular Mn/Fe ratio in streptococci could result in higher percentages of PerR:Zn,Mn than of the PerR:Zn,Fe found in bacilli; thus, cysteine oxidation contributes to H_2_O_2_ inactivation of the streptococcal PerR:Zn,Mn proteins (Fig. 4 and 5; Table S1). Nevertheless, the possibility of Fe^2+^-triggered streptococcal PerR inactivation cannot be excluded, as approximately 28% of His40 residues and 53% of His95 residues in PerR were oxidized when S. oligofermentans was grown in BHI broth containing 0.5 μM Mn^2+^ and 15 μM Fe^2+^ (Table S1). Therefore, the dual-H_2_O_2_-sensing mechanisms of the redox regulator PerR could provide protection for the catalase-negative streptococci from oxidative stress in environments with different metal ions.

10.1128/mSystems.00006-20.5 FIG S5 Sequence alignment and structural comparison of Zn^2+^-coordinated cysteine residues of the PerR proteins from S. oligofermentans and B. subtilis. (A) The amino acid sequences of the S. oligofermentans (UniProt accession number A0A512AEK5) and B. subtilis (UniProt accession number P71086) PerRs were retrieved from the UniProt protein database. Sequence alignment was performed using the DNAMAN program. *, conserved amino acid residues essential for manganese or ferric ion binding in B. subtilis; #, conserved cysteine residues for zinc ion binding. Black shadings indicate 100% homology. (B) Structural homology modeling of the S. oligofermentans PerR, performed using the SWISS-MODEL server by automatically selecting S. pyogenes PerR (PDB accession number 4LMY) as a template (left) and the crystal structure of B. subtilis PerR (PDB accession number 2FE3) (right). Four Zn^2+^-coordinated cysteine residues are shown as sticks, and the corresponding cysteine residues are labeled. Download FIG S5, TIF file, 2.1 MB. Copyright © 2020 Tong et al. 2020 Tong et al. This content is distributed under the terms of the Creative Commons Attribution 4.0 International license.

Metal homeostasis plays a central role in oxidative stress resistance in Gram-positive bacteria (4, 44, 47, 48). In addition to PerR, streptococci also employ MntR, a metalloregulator protein, to control cellular manganese and iron homeostasis (11, 16, 21, 25). Oxidative inactivation of PerR and MntR derepresses the expression of the metal homeostasis-related genes *dpr* and *mntABC* (Table 3) (25). Dpr chelates cellular Fe^2+^ and so prevents the production of highly toxic HO·, and the manganese importer MntABC takes up Mn^2+^ to decompose cellular H_2_O_2_. MntABC and Dpr have been verified to protect S. oligofermentans from challenge by a high H_2_O_2_ concentration in our previous studies (11). Here, Dpr was further verified to resist a sublethal H_2_O_2_ concentration.

The thioredoxin (Trx) system, which is comprised of NADPH, thioredoxin reductase (TrxR), and thioredoxin, plays a key role in defense against oxidative stress, particularly in the catalase-lacking streptococci (9, 49–51). Oxidation of the cysteine thiol groups of Trx and Tpx has been found in 40 μM H_2_O_2_-pretreated S. oligofermentans cells (Fig. 3), and deletion of two genes increases the H_2_O_2_ sensitivity of the streptococcus (Fig. 6). This observation indicates that the Trx system is involved in the H_2_O_2_ adaptation of S. oligofermentans, which is presumably under the control of PerR.

In conclusion, this work reports a novel H_2_O_2_ adaptation mechanism. Trace amounts of cellular H_2_O_2_ cause thiol oxidation of the redox-based regulatory and functional proteins and activate antioxidative systems; meanwhile, they reduce the level of glycolysis, which generates ROS precursors. This H_2_O_2_ adaptation mechanism could be an important antioxidative defense strategy of the catalase-void anaerobes.

## MATERIALS AND METHODS

### Bacterial strains and culture conditions.

S. oligofermentans AS 1.3089 (52) and its derivative strains (see Table S3 in the supplemental material) were grown in brain heart infusion (BHI) broth (BD Difco, Franklin Lakes, NJ) statically or anaerobically under 100% N_2_. Escherichia coli DH5α, used for cloning, was grown in Luria-Bertani (LB) broth at 37°C under shaking. When required, kanamycin (1 mg/ml) and spectinomycin (1 mg/ml) were used for the selection of *Streptococcus* transformants, while ampicillin (100 μg/ml) and spectinomycin (250 μg/ml) were used to select E. coli transformants.

10.1128/mSystems.00006-20.8 TABLE S3 Strains, plasmids, and primers used in this study. *, Amp, ampicillin; Kan, kanamycin; Sp, spectinomycin; r, resistant; s, sensitive. Italic nucleotide bases indicate restriction enzyme digestion sites. Download Table S3, DOCX file, 0.02 MB. Copyright © 2020 Tong et al. 2020 Tong et al. This content is distributed under the terms of the Creative Commons Attribution 4.0 International license.

### Construction of genetically altered strains.

All primers used in this study are listed in Table S3. *tpx*, *trx*, *dpr*, and *perR* deletion strains were constructed using the PCR-ligation method (53). The upstream and downstream DNA fragments of each gene were amplified from the genomic DNA. The purified, BamHI-digested PCR products were ligated with a kanamycin resistance gene fragment from plasmid pALH124 (54) or a spectinomycin resistance gene fragment from pDL278 (55) at compatible sites. For construction of 6×His-tagged strains, the *tpx*, *trx*, and *perR* genes were amplified from the genomic DNA using a pair of primers, with the reverse primer carrying a sequence encoding 6 histidines just before the termination codon. Meanwhile, an ∼600-bp DNA fragment immediately downstream of the termination codon of each gene was amplified. The purified PCR products were digested with BamHI and ligated with the kanamycin resistance gene fragment. The ligation mixtures were transformed into the S. oligofermentans wild-type strain, except that the ligation mixture for *perR* deletion was transformed into the Δ*mntR* strain (11) to construct a Δ*perR* Δ*mntR* strain, as described previously (6). For construction of the *perR*::pDL278-*perR*C139S-6×His and *perR*::pDL278-*perR*C142S-6×His strains, the *perR*-6×His gene fusion was amplified from the genomic DNA of the strain with 6×His-tagged PerR. After digestion with EcoRI and SalI, the purified product was inserted into the compatible sites on the E. coli-streptococci shuttle vector pDL278 (55) to produce pDL278-*perR*-6×His. Then, Cys139 and Cys142 were mutated into serine using a site-directed gene mutagenesis kit (Beyotime Biotechnology Co., Shanghai, China). The correct pDL278-*perR*-6×His, pDL278-*perR*C139S-6×His, and pDL278-*perR*C142S-6×His plasmids were transformed into the *perR* deletion strain to produce strains ectopically expressing wild-type and cysteine-mutated *perR*.

### Detection of intracellular hydrogen peroxide by HyPer imaging.

Mid-exponential-phase HyPer reporter cells were pelleted, washed twice with phosphate-buffered saline (PBS), resuspended in 100 μl of PBS, and exposed to air in the dark for 30 min. Forty microliters of cells was placed on a Polysine microscope slide (25 by 75 by 1 mm; Thermo Scientific, Waltham, MA), covered with a Fisher-brand microscope glass coverslip (diameter, 15 mm; thickness, 0.13 to 0.17 mm; Thermo Scientific), and then visualized under a confocal laser scanning microscope (Leica model TCS SP8; Leica Microsystems, Buffalo Grove, IL, USA). Excitation was provided at 488 nm, with emission being collected from a wavelength range of 500 to 530 nm (32, 56). For each sample, at least 5 fluorescent and differential interference contrast (DIC) images were captured. The fluorescence intensities of 25 regions of interest (ROI), with each ROI containing 5 cells, from each sample were measured using Leica Application Suite (LAS) AF software. For images with fluorescence that was too weak, the ROI in the corresponding DIC images was framed, and the fluorescence was measured in the same ROI in the fluorescence image. The average fluorescence intensities of 25 ROIs were calculated and are expressed in arbitrary units (a.u.) per ROI ± standard deviation.

### Redox proteomics analysis by differential alkylation and LC-MS/MS.

The differential alkylation method (34) was used to identify H_2_O_2_-induced changes in the thiol redox status of the proteins. Briefly, mid-exponential-phase cells in the tested samples were collected by centrifugation and resuspended in radioimmunoprecipitation assay (RIPA) buffer (50 mM Tris [pH 7.4], 150 mM NaCl, 1% Triton X-100, 1% sodium deoxycholate, 0.1% SDS, 2 mM sodium pyrophosphate, 25 mM β-glycerophosphate, 1 mM sodium orthovanadate, sodium fluoride, 1 mM EDTA, 0.5 μg/ml leupeptin) containing 1 mM phenylmethylsulfonyl fluoride (PMSF). To minimize an artificial oxidation during sample preparation, cell breakage by sonication was performed inside an anaerobic chamber (Thermo Scientific); moreover, 10 mM EDTA and 1 kU/ml catalase were included in the lysis buffer to prevent an Fe^2+^-triggered Fenton reaction and decompose H_2_O_2_, respectively. The sonication was implemented on ice in the dark using a UP-400S ultrasonicator (Xinzi Company, Ningbo, China), and cell lysates were centrifuged at 8,000 × *g* for 15 min, and then the protein concentration in the supernatant was measured using a Pierce bicinchoninic acid (BCA) protein assay kit (Thermo Scientific). The same amounts of protein from all the samples were separated on a nonreducing one-dimensional SDS-PAGE gel, and each gel lane was cut into 6 slices and washed with MS-grade water three times. The proteins in the gel were alkylated for 30 min with 55 mM [^13^C]iodoacetic acid in 50 mM NH_4_HCO_3_ (pH 8.0) in the dark. After removing the iodoacetic acid, 25 mM DTT reduction was performed for 45 min at 55°C, and then the DTT was removed and the proteins were alkylated with 55 mM [^12^C]iodoacetic acid for 30 min in the dark. Upon in-gel digestion with MS-grade trypsin (Promega, Fitchburg, WI), LC-MS/MS analysis was implemented with an Easy-nLC integrated nano-high-performance liquid chromatography system (Proxeon, Odense, Denmark) and a Q-Extractive mass spectrometer (Thermo Scientific, Waltham, MA), as described previously (28).

MS/MS spectra were searched against the forward and reverse S. oligofermentans protein database, downloaded from UniProt, using the SEQUEST search engine of Proteome Discoverer software (v1.4). The precursor ion mass tolerance was 20 ppm for all mass spectra acquired in an Orbitrap mass analyzer, and the fragment ion mass tolerance was 0.02 Da for all MS/MS spectra. The following search criteria were employed: full tryptic specificity was required; two missed cleavages were allowed; ^13^C carboxymethylation (free cysteine residue), ^12^C carboxymethylation (disulfide linkage cysteine residue) and sulfenic, sulfinic, and sulfonic acids were variable modifications for cysteine; oxidation was a variable modification for methionine; and the false discovery rate (FDR) was set to 0.01. All the cysteine-modified MS/MS spectra were manually confirmed. The MaxQuant software package was used to obtain the intensity of the cysteine-modified peptides. Duplicate experiments were performed in parallel.

### Protein GO category analysis.

Homologues of the S. oligofermentans redox-sensitive proteins were searched for in S. pneumoniae and put into the PANTHER bioinformatics platform (http://www.pantherdb.org/) for Gene Ontology (GO) analysis. GO enrichment analysis was implemented on the Gene Ontology Consortium website (http://www.geneontology.org), the binomial test was used for analysis of statistical significance, and a *P* value of <0.05 was used as a cutoff.

### Redox Western blotting.

Cells were collected by centrifugation and resuspended in RIPA buffer (50 mM Tris-HCl, pH 7.4, 150 mM NaCl, 1% Triton X-100, 1% sodium deoxycholate, 0.1% SDS, sodium orthovanadate, sodium fluoride, EDTA, leupeptin) with addition of 40 mM *N*-ethylmaleimide (NEM), 1 mM PMSF, 10 mM EDTA, and 1 kU/ml catalase. Cells were sonicated on ice in the dark for 45 min and alkylated in the dark for 20 min, and then the supernatant were collected by centrifugation. Reduced samples were prepared by incubating the lysates with 50 mM DTT for 1 h. For the 4-acetamido-4′-maleimidylstilbene-2,2′-disulfonic acid (AMS) alkylating experiment, cells were resuspended in PBS buffer containing 15 mM AMS, 1 mM PMSF, 10 mM EDTA, and 1 kU/ml catalase, sonicated, and then incubated at 4°C for 2 h in the dark. Half of the samples were reduced with 50 mM DTT for 1 h, and then the DTT was removed and the samples were alkylated with 15 mM AMS at 4°C for 2 h in the dark. The protein concentration of the lysate was determined using a BCA protein assay kit. Protein samples were diluted in nonreducing loading buffer (4×; 0.2 M Tris-HCl, pH 6.8, 40% glycerol, 8% SDS, 0.4% bromphenol blue), separated by SDS-PAGE, transferred onto a nitrocellulose membrane, and hybridized with an anti-His tag antibody (Abmart Company, Shanghai, China) at a 4,000-fold dilution. Detection was performed using a chemiluminescent nucleic acid detection module kit (Thermo Scientific).

### IP and LC-MS/MS identification of cysteine thiol oxidation of the PerR protein *in vivo*.

6×His-tagged PerR protein was purified by immunoprecipitation (IP) using anti-His tag monoclonal antibody-magnetic agarose (MBL International Corporation, Woburn, MA) according to the instructions of the manufacturer. Briefly, the 6×His-tagged-PerR-expressing strain PerR-6×His was statically grown in BHI broth. The mid-exponential-phase cells were collected and washed with PBS three times. Then, the cells were resuspended in lysis buffer (50 mM Tris-HCl, 150 mM NaCl, 0.05% NP-40, 1 mM DTT) containing 55 mM [^13^C]iodoacetic acid, 10 mM EDTA and 1 kU/ml catalase. The cells were sonicated on ice in the dark for 45 min and alkylated in the dark for 20 min, and then the cell lysate was subjected to centrifugation. The obtained supernatant was mixed and incubated with the magnetic beads. After washing 4 times with lysis buffer, the immunoprecipitated 6×His-tagged PerR protein was eluted by boiling in nonreducing SDS sample buffer (4% SDS, 125 mM Tris-HCl, pH 8.0, 20% glycerol) and separated using 18% nonreducing SDS-PAGE. The target PerR protein band with the expected molecular size was cut from the gel, and cysteine residue oxidation was identified by differential alkylation and LC-MS/MS, as described above, except that the reduced and oxidized cysteine residues were alkylated with 55 mM [^13^C]iodoacetic acid and [^12^C]iodoacetamide, respectively.

### Overexpression of PerR-GST, Tpx-6×His, and Trx-6×His proteins.

A 450-bp DNA fragment containing the entire *perR* coding gene was PCR amplified. The purified PCR product was digested with EcoRI/XhoI and ligated into the compatible sites on pGEX4T-1 (GE Healthcare, Boston, MA), and the produced pGEX-PerR was transformed into E. coli BL21(DE3) cells (Novagen, Madison, WI). Correct transformants were grown at 37°C to an OD_600_ of 0.4 to 0.6, and 0.1 mM isopropyl-β-d-thiogalactopyranoside (IPTG; Sigma-Aldrich, St. Louis, MO) was added to induce PerR-GST expression at 22°C overnight. Then, the cells were collected by centrifugation and resuspended in phosphate-buffered saline (PBS; 10 mM Na_2_HPO_4_, 1.8 mM KH_2_PO_4_, 137 mM NaCl, 2.7 mM KCl, pH 7.4) containing 1 mM DTT and 10 mM EDTA and then lysed by sonication for 30 min. The cell lysate was centrifuged at 8,000 × *g* for 30 min, and the supernatant was filtered through a 0.22-nm-pore-size polyvinylidene difluoride membrane (Millipore, Billerica, MA) and then applied to a GSTrap HP column (GE Healthcare, Boston, MA). The proteins were eluted with elution buffer (20 mM Tris-HCl buffer containing 1 mM DTT, 10 mM EDTA, and 10 mM reduced glutathione [GSH], pH 8.0), and the elution fractions were analyzed by electrophoresis on a 12% SDS-PAGE gel. The fractions with the desired protein were pooled and dialyzed against PBS buffer containing 3 mM GSH and 10 mM EDTA three times. Then, the purified proteins were stored in aliquots in 10% glycerol at −80°C until use.

For the overexpression of the Tpx-6×His and Trx-6×His proteins, 492- and 552-bp DNA fragments containing the entire *tpx* and *trx* coding genes, respectively, were PCR amplified with the primer pairs listed in Table S3. The resultant products were integrated into pET-28a (Novagen, Madison, WI) by Gibson assembly (New England Biolabs, Beverly, MA) to produce pET-28a-Tpx and pET-28a-Trx. The correct constructs were transformed into E. coli BL21(DE3) (Novagen, Madison, WI) cells. Correct transformants were grown at 37°C to an OD_600_ of 0.6 to 0.8, 0.1 mM IPTG (Sigma-Aldrich, St. Louis, MO) was added, and the cells were incubated at 22°C overnight. Then, the cells were collected by centrifugation, resuspended in binding buffer (20 mM sodium phosphate, 500 mM NaCl, 30 mM imidazole, 1 mM EDTA, 1 mM DTT, pH 7.4), and lysed by sonication for 30 min. The supernatant was filtered and then applied to an Ni^2+^-charged chelating column (GE Healthcare, Piscataway, NJ) that had previously been equilibrated with binding buffer. Proteins were eluted with elution buffer (20 mM sodium phosphate, 500 mM NaCl, 500 mM imidazole, 1 mM DTT, pH 7.4). The fractions with the desired protein were pooled and dialyzed against buffer containing 20 mM Tris-HCl, 150 mM NaCl, 1 mM DTT, and 1 mM EDTA. The purified Tpx-6×His and Trx-6×His proteins were stored in aliquots in 10% glycerol at −80°C until use.

### Nonreducing SDS-PAGE.

Five micrograms of PerR-GST protein was treated or not treated with 5 mM H_2_O_2_ for 30 min and with or without a subsequent reduction by 50 mM DTT for 1 h. Before electrophoresis, 40 mM NEM was added, and the mixture was kept in the dark for 30 min. The protein samples were diluted in nonreducing SDS loading buffer (4×; 0.2 M Tris-HCl, pH 6.8, 40% glycerol, 8% SDS, 0.4% bromphenol blue) and then separated on a 12% SDS-PAGE gel.

### Determination of zinc content in PerR-GST using ICP-MS.

The PerR-GST protein was treated or not treated with 5 mM H_2_O_2_ for 30 min and with or without a subsequent reduction by 50 mM DTT for 1 h and was then transferred into Chelex 100-treated PBS buffer via ultrafiltration. Protein concentrations were measured with a BCA protein assay kit. The protein samples were treated with nitric acid (ultrapure), and then the zinc content was analyzed by inductively coupled plasma mass spectrometry (ICP-MS; DRCII apparatus; PerkinElmer, USA) at Peking University Health Science Center. Beryllium, indium, and uranium standard solutions (NIST certified; PerkinElmer) were used to calibrate the ICP-MS. Experiments were conducted for triplicate samples and repeated at least three times.

### Electrophoretic mobility shift assay (EMSA).

The target gene promoter fragments were generated by PCR amplification using the biotin-labeled primer pair listed in Table S3. The PerR-GST protein was first dialyzed into PBS buffer containing 10 mM EDTA and 1 mM DTT and then digested with 100 U thrombin to remove the GST tag. One micromole of the PerR protein was preincubated with 1 mM MnCl_2_, and then 0.2 nM a biotin-labeled double-stranded DNA probe and increasing amounts of PerR (0 to 200 nM) were mixed in the binding buffer [10 mM Tris-HCl, pH 8.0, 5% glycerol, 50 mM NaCl, 10 μg/ml bovine serum albumin, 2 ng/μl poly(dI·dC), 0.5 mM DTT, 1 mM MnCl_2_]. The reaction proceeded at 30°C for 30 min. To observe the effect of H_2_O_2_ on PerR binding, 1 μM PerR protein was preincubated with 1 mM MnCl_2_ and then treated with various concentrations of H_2_O_2_ (0 to 200 μM) at 30°C for 30 min or with 200 μM H_2_O_2_ at 30°C for various times (0 to 30 min), and then catalase was added to a final concentration of 150 U/ml and the mixture was incubated at 37°C for 30 min. To determine whether oxidation was reversible, 10 mM DTT was added to reduce the 200 μM H_2_O_2_-treated PerR at 37°C for 1 h. Then, 50 nM H_2_O_2_-oxidized or DTT-reduced PerR protein was tested for binding to a 0.2 nM biotin-labeled *dpr* promoter fragment. The binding mixtures were electrophoresed on a 6% polyacrylamide gel on ice. The DNA-protein complex was transferred onto a nylon membrane and detected with a chemiluminescent nucleic acid detection module kit (Thermo Scientific).

### Determination of H_2_O_2_ survival rate.

Overnight cultures of the tested strains were diluted 1:30 into fresh BHI broth and incubated strictly anaerobically. When the OD_600_ reached 0.4 to 0.5, the cells were separated into three aliquots. One aliquot was treated with 10 mM H_2_O_2_ for 10 min, and another was prepulsed with 40 μM H_2_O_2_ for 20 min before being subjected to 10 mM H_2_O_2_ treatment, while an aliquot not treated with 10 mM H_2_O_2_ was used as a control. Then, the cells were collected, washed twice with PBS, and resuspended in 200 μl BHI broth. Cell chains were separated by sonication for 30 s with an XC-3200D ultrasonic cleaner (Xinchen Company, Nanjing, China), and then 10-fold serial dilutions were performed. Appropriate dilutions were plated on BHI agar plates, and the numbers of CFU were counted after 24 h of incubation in a candle jar at 37°C. The survival percentage was calculated by dividing the number of CFU of the H_2_O_2_-challenged sample by the number of CFU of the corresponding controls. Experiments were executed in triplicate, and each experiment was repeated at least three times independently.

### Assay of growth under H_2_O_2_ stress.

S. oligofermentans wild-type and gene deletion strains were grown anaerobically in BHI broth until the OD_600_ reached ∼0.5, with three replicates of each strain being included. Two replicate cultures were supplemented with 40 and 100 μM H_2_O_2_, respectively, leaving one replicate as an H_2_O_2_-untreated control. The growth profiles were measured by counting the number of CFU at the different time intervals. Triplicates for each sample were measured, and the experiments were repeated at least three times.

### Determination of excreted hydrogen peroxide in culture.

The hydrogen peroxide in the culture suspension was quantified as described previously (11). Briefly, 650 μl of culture supernatant was added to 600 μl of a solution containing 2.5 mM 4-amino-antipyrine (4-amino-2,3-dimethyl-1-phenyl-3-pyrazolin-5-one) (Sigma-Aldrich) and 0.17 M phenol. The reaction proceeded for 4 min at room temperature; horseradish peroxidase (Sigma-Aldrich) was then added to a final concentration of 50 mU/ml in 0.2 M potassium phosphate buffer (pH 7.2). After 4 min of incubation at room temperature, the optical density at 510 nm was measured with a Unico 2100 visible spectrophotometer (Unico, Shanghai, China). A standard curve was generated with known concentrations of chemical H_2_O_2_.

### Quantitative PCR.

Total RNA was extracted from mid-exponential-phase (OD_600_, ∼0.4 to 0.5) H_2_O_2_-treated and -untreated S. oligofermentans cells using the TRIzol reagent (Invitrogen, Carlsbad, CA), as recommended by the supplier. After quality confirmation with a 1% agarose gel, the RNA was treated with RNase-free DNase (Promega, Madison, WI) and analyzed by PCR for possible chromosomal DNA contamination. cDNA was generated from 2 μg total RNA with random primers using Moloney murine leukemia virus reverse transcriptase (Promega, Madison, WI), according to the supplier’s instructions, and was used for quantitative PCR (qPCR) amplification with the corresponding primers (Table S3). Amplifications were performed with a Mastercycler ep realplex^2^ instrument (Eppendorf, Germany). To estimate the copy numbers of the tested genes, a standard curve for each tested gene was generated by quantitative PCR using a 10-fold serially diluted PCR product as the template. The 16S rRNA gene was used as the biomass reference. The number of copies of the tested gene transcript per 100 16S rRNA copies is shown. All measurements were done for triplicate samples, and the experiments were repeated at least three times.

## References

[1] Miller RA, Britigan BE 1997 Role of oxidants in microbial pathophysiology. Clin Microbiol Rev 10:1–18. doi:10.1128/CMR.10.1.1. 8993856 PMC172912

[2] Winterbourn CC, Kettle AJ, Hampton MB 2016 Reactive oxygen species and neutrophil function. Annu Rev Biochem 85:765–792. doi:10.1146/annurev-biochem-060815-014442. 27050287

[3] Imlay JA 2013 The molecular mechanisms and physiological consequences of oxidative stress: lessons from a model bacterium. Nat Rev Microbiol 11:443–454. doi:10.1038/nrmicro3032. 23712352 PMC4018742

[4] Faulkner MJ, Helmann JD 2011 Peroxide stress elicits adaptive changes in bacterial metal ion homeostasis. Antioxid Redox Signal 15:175–189. doi:10.1089/ars.2010.3682. 20977351 PMC3110094

[5] Liu L, Tong H, Dong X 2012 Function of the pyruvate oxidase-lactate oxidase cascade in interspecies competition between *Streptococcus oligofermentans* and *Streptococcus mutans*. Appl Environ Microbiol 78:2120–2127. doi:10.1128/AEM.07539-11. 22287002 PMC3302633

[6] Tong H, Chen W, Merritt J, Qi F, Shi W, Dong X 2007 *Streptococcus oligofermentans* inhibits *Streptococcus mutans* through conversion of lactic acid into inhibitory H_2_O_2_: a possible counteroffensive strategy for interspecies competition. Mol Microbiol 63:872–880. doi:10.1111/j.1365-2958.2006.05546.x. 17302806

[7] Tong H, Chen W, Shi W, Qi F, Dong X 2008 SO-LAAO, a novel l-amino acid oxidase that enables *Streptococcus oligofermentans* to outcompete *Streptococcus mutans* by generating H_2_O_2_ from peptone. J Bacteriol 190:4716–4721. doi:10.1128/JB.00363-08. 18469105 PMC2446784

[8] Lisher JP, Tsui HT, Ramos-Montanez S, Hentchel KL, Martin JE, Trinidad JC, Winkler ME, Giedroc DP 2017 Biological and chemical adaptation to endogenous hydrogen peroxide production in *Streptococcus pneumoniae* D39. mSphere 2:e00291-16. doi:10.1128/mSphere.00291-16. PMC5214746 28070562

[9] Henningham A, Dohrmann S, Nizet V, Cole JN 2015 Mechanisms of group A *Streptococcus* resistance to reactive oxygen species. FEMS Microbiol Rev 39:488–508. doi:10.1093/femsre/fuu009. 25670736 PMC4487405

[10] Yesilkaya H, Andisi VF, Andrew PW, Bijlsma JJ 2013 *Streptococcus pneumoniae* and reactive oxygen species: an unusual approach to living with radicals. Trends Microbiol 21:187–195. doi:10.1016/j.tim.2013.01.004. 23415028

[11] Wang X, Tong H, Dong X 2014 PerR-regulated manganese ion uptake contributes to oxidative stress defense in an oral streptococcus. Appl Environ Microbiol 80:2351–2359. doi:10.1128/AEM.00064-14. 24487543 PMC3993191

[12] Hillion M, Antelmann H 2015 Thiol-based redox switches in prokaryotes. Biol Chem 396:415–444. doi:10.1515/hsz-2015-0102. 25720121 PMC4438307

[13] Antelmann H, Helmann JD 2011 Thiol-based redox switches and gene regulation. Antioxid Redox Signal 14:1049–1063. doi:10.1089/ars.2010.3400. 20626317 PMC3113447

[14] Marinho HS, Real C, Cyrne L, Soares H, Antunes F 2014 Hydrogen peroxide sensing, signaling and regulation of transcription factors. Redox Biol 2:535–562. doi:10.1016/j.redox.2014.02.006. 24634836 PMC3953959

[15] Zheng M, Aslund F, Storz G 1998 Activation of the OxyR transcription factor by reversible disulfide bond formation. Science 279:1718–1721. doi:10.1126/science.279.5357.1718. 9497290

[16] Turner AG, Ong CY, Djoko KY, West NP, Davies MR, McEwan AG, Walker MJ 2017 The PerR-regulated P_1B-4_-type ATPase (PmtA) acts as a ferrous iron efflux pump in *Streptococcus pyogenes*. Infect Immun 85:e00140-17. doi:10.1128/IAI.00140-17. 28373352 PMC5442638

[17] Fuangthong M, Herbig AF, Bsat N, Helmann JD 2002 Regulation of the *Bacillus subtilis fur* and *perR* genes by PerR: not all members of the PerR regulon are peroxide inducible. J Bacteriol 184:3276–3286. doi:10.1128/jb.184.12.3276-3286.2002. 12029044 PMC135084

[18] Jacquamet L, Traore DA, Ferrer JL, Proux O, Testemale D, Hazemann JL, Nazarenko E, El Ghazouani A, Caux-Thang C, Duarte V, Latour JM 2009 Structural characterization of the active form of PerR: insights into the metal-induced activation of PerR and Fur proteins for DNA binding. Mol Microbiol 73:20–31. doi:10.1111/j.1365-2958.2009.06753.x. 19508285

[19] Herbig AF, Helmann JD 2001 Roles of metal ions and hydrogen peroxide in modulating the interaction of the *Bacillus subtilis* PerR peroxide regulon repressor with operator DNA. Mol Microbiol 41:849–859. doi:10.1046/j.1365-2958.2001.02543.x. 11532148

[20] Lee JW, Helmann JD 2006 The PerR transcription factor senses H_2_O_2_ by metal-catalysed histidine oxidation. Nature 440:363–367. doi:10.1038/nature04537. 16541078

[21] Makthal N, Rastegari S, Sanson M, Ma Z, Olsen RJ, Helmann JD, Musser JM, Kumaraswami M 2013 Crystal structure of peroxide stress regulator from *Streptococcus pyogenes* provides functional insights into the mechanism of oxidative stress sensing. J Biol Chem 288:18311–18324. doi:10.1074/jbc.M113.456590. 23645680 PMC3689973

[22] Grifantini R, Toukoki C, Colaprico A, Gryllos I 2011 Peroxide stimulon and role of PerR in group A *Streptococcus*. J Bacteriol 193:6539–6551. doi:10.1128/JB.05924-11. 21949080 PMC3232902

[23] Rhee SG 2006 Cell signaling. H_2_O_2_, a necessary evil for cell signaling. Science 312:1882–1883. doi:10.1126/science.1130481. 16809515

[24] Sun F, Liang H, Kong X, Xie S, Cho H, Deng X, Ji Q, Zhang H, Alvarez S, Hicks LM, Bae T, Luo C, Jiang H, He C 2012 Quorum-sensing agr mediates bacterial oxidation response via an intramolecular disulfide redox switch in the response regulator AgrA. Proc Natl Acad Sci U S A 109:9095–9100. doi:10.1073/pnas.1200603109. 22586129 PMC3384213

[25] Chen Z, Wang X, Yang F, Hu Q, Tong H, Dong X 2017 Molecular insights into hydrogen peroxide-sensing mechanism of the metalloregulator MntR in controlling bacterial resistance to oxidative stresses. J Biol Chem 292:5519–5531. doi:10.1074/jbc.M116.764126. 28223356 PMC5392694

[26] Lindahl M, Mata-Cabana A, Kieselbach T 2011 The disulfide proteome and other reactive cysteine proteomes: analysis and functional significance. Antioxid Redox Signal 14:2581–2642. doi:10.1089/ars.2010.3551. 21275844

[27] Leonard SE, Carroll KS 2011 Chemical ‘omics’ approaches for understanding protein cysteine oxidation in biology. Curr Opin Chem Biol 15:88–102. doi:10.1016/j.cbpa.2010.11.012. 21130680

[28] Wang J, Jin L, Li X, Deng H, Chen Y, Lian Q, Ge R, Deng H 2013 Gossypol induces apoptosis in ovarian cancer cells through oxidative stress. Mol Biosyst 9:1489–1497. doi:10.1039/c3mb25461e. 23532321

[29] Leichert LI, Gehrke F, Gudiseva HV, Blackwell T, Ilbert M, Walker AK, Strahler JR, Andrews PC, Jakob U 2008 Quantifying changes in the thiol redox proteome upon oxidative stress in vivo. Proc Natl Acad Sci U S A 105:8197–8202. doi:10.1073/pnas.0707723105. 18287020 PMC2448814

[30] Deng X, Weerapana E, Ulanovskaya O, Sun F, Liang H, Ji Q, Ye Y, Fu Y, Zhou L, Li J, Zhang H, Wang C, Alvarez S, Hicks LM, Lan L, Wu M, Cravatt BF, He C 2013 Proteome-wide quantification and characterization of oxidation-sensitive cysteines in pathogenic bacteria. Cell Host Microbe 13:358–370. doi:10.1016/j.chom.2013.02.004. 23498960 PMC3652280

[31] Pericone CD, Park S, Imlay JA, Weiser JN 2003 Factors contributing to hydrogen peroxide resistance in *Streptococcus pneumoniae* include pyruvate oxidase (SpxB) and avoidance of the toxic effects of the Fenton reaction. J Bacteriol 185:6815–6825. doi:10.1128/jb.185.23.6815-6825.2003. 14617646 PMC262707

[32] Belousov VV, Fradkov AF, Lukyanov KA, Staroverov DB, Shakhbazov KS, Terskikh AV, Lukyanov S 2006 Genetically encoded fluorescent indicator for intracellular hydrogen peroxide. Nat Methods 3:281–286. doi:10.1038/nmeth866. 16554833

[33] Tong H, Wang X, Dong Y, Hu Q, Zhao Z, Zhu Y, Dong L, Bai F, Dong X 2019 A *Streptococcus* aquaporin acts as peroxiporin for efflux of cellular hydrogen peroxide and alleviation of oxidative stress. J Biol Chem 294:4583–4595. doi:10.1074/jbc.RA118.006877. 30705089 PMC6433050

[34] Schilling B, Yoo CB, Collins CJ, Gibson BW 2004 Determining cysteine oxidation status using differential alkylation. Int J Mass Spectrom 236:117–127. doi:10.1016/j.ijms.2004.06.004.

[35] Mi H, Huang X, Muruganujan A, Tang H, Mills C, Kang D, Thomas PD 2017 PANTHER version 11: expanded annotation data from Gene Ontology and Reactome pathways, and data analysis tool enhancements. Nucleic Acids Res 45:D183–D189. doi:10.1093/nar/gkw1138. 27899595 PMC5210595

[36] Hajaj B, Yesilkaya H, Benisty R, David M, Andrew PW, Porat N 2012 Thiol peroxidase is an important component of *Streptococcus pneumoniae* in oxygenated environments. Infect Immun 80:4333–4343. doi:10.1128/IAI.00126-12. 23027531 PMC3497430

[37] Jakob U, Eser M, Bardwell JC 2000 Redox switch of Hsp33 has a novel zinc-binding motif. J Biol Chem 275:38302–38310. doi:10.1074/jbc.M005957200. 10976105

[38] Bae JB, Park JH, Hahn MY, Kim MS, Roe JH 2004 Redox-dependent changes in RsrA, an anti-sigma factor in *Streptomyces coelicolor*: zinc release and disulfide bond formation. J Mol Biol 335:425–435. doi:10.1016/j.jmb.2003.10.065. 14672653

[39] Horsburgh MJ, Clements MO, Crossley H, Ingham E, Foster SJ 2001 PerR controls oxidative stress resistance and iron storage proteins and is required for virulence in *Staphylococcus aureus*. Infect Immun 69:3744–3754. doi:10.1128/IAI.69.6.3744-3754.2001. 11349039 PMC98383

[40] Hillmann F, Doring C, Riebe O, Ehrenreich A, Fischer RJ, Bahl H 2009 The role of PerR in O_2_-affected gene expression of *Clostridium acetobutylicum*. J Bacteriol 191:6082–6093. doi:10.1128/JB.00351-09. 19648241 PMC2747897

[41] Di Simplicio P, Franconi F, Frosali S, Di Giuseppe D 2003 Thiolation and nitrosation of cysteines in biological fluids and cells. Amino Acids 25:323–339. doi:10.1007/s00726-003-0020-1. 14661094

[42] Hahn JS, Oh SY, Chater KF, Cho YH, Roe JH 2000 H_2_O_2_-sensitive Fur-like repressor CatR regulating the major catalase gene in *Streptomyces coelicolor*. J Biol Chem 275:38254–38260. doi:10.1074/jbc.M006079200. 10991944

[43] Cremers CM, Jakob U 2013 Oxidant sensing by reversible disulfide bond formation. J Biol Chem 288:26489–26496. doi:10.1074/jbc.R113.462929. 23861395 PMC3772196

[44] Helmann JD 2014 Specificity of metal sensing: iron and manganese homeostasis in *Bacillus subtilis*. J Biol Chem 289:28112–28120. doi:10.1074/jbc.R114.587071. 25160631 PMC4192466

[45] Lisher JP, Giedroc DP 2013 Manganese acquisition and homeostasis at the host-pathogen interface. Front Cell Infect Microbiol 3:91. doi:10.3389/fcimb.2013.00091. 24367765 PMC3851752

[46] Imlay JA 2019 Where in the world do bacteria experience oxidative stress? Environ Microbiol 21:521–530. doi:10.1111/1462-2920.14445. 30307099 PMC7301649

[47] Aguirre JD, Culotta VC 2012 Battles with iron: manganese in oxidative stress protection. J Biol Chem 287:13541–13548. doi:10.1074/jbc.R111.312181. 22247543 PMC3340200

[48] Turner AG, Ong CL, Gillen CM, Davies MR, West NP, McEwan AG, Walker MJ 2015 Manganese homeostasis in group A *Streptococcus* is critical for resistance to oxidative stress and virulence. mBio 6:e00278-15. doi:10.1128/mBio.00278-15. 25805729 PMC4453566

[49] Rhodes DV, Crump KE, Makhlynets O, Snyder M, Ge X, Xu P, Stubbe J, Kitten T 2014 Genetic characterization and role in virulence of the ribonucleotide reductases of *Streptococcus sanguinis*. J Biol Chem 289:6273–6287. doi:10.1074/jbc.M113.533620. 24381171 PMC3937693

[50] Xu Y, Itzek A, Kreth J 2014 Comparison of genes required for H_2_O_2_ resistance in *Streptococcus gordonii* and *Streptococcus sanguinis*. Microbiology 160:2627–2638. doi:10.1099/mic.0.082156-0. 25280752 PMC4252910

[51] Marco S, Rullo R, Albino A, Masullo M, De Vendittis E, Amato M 2013 The thioredoxin system in the dental caries pathogen *Streptococcus mutans* and the food-industry bacterium *Streptococcus thermophilus*. Biochimie 95:2145–2156. doi:10.1016/j.biochi.2013.08.008. 23954859

[52] Tong H, Gao X, Dong X 2003 *Streptococcus oligofermentans* sp. nov., a novel oral isolate from caries-free humans. Int J Syst Evol Microbiol 53:1101–1104. doi:10.1099/ijs.0.02493-0. 12892133

[53] Lau PC, Sung CK, Lee JH, Morrison DA, Cvitkovitch DG 2002 PCR ligation mutagenesis in transformable streptococci: application and efficiency. J Microbiol Methods 49:193–205. doi:10.1016/s0167-7012(01)00369-4. 11830305

[54] Liu Y, Zeng L, Burne RA 2009 AguR is required for induction of the *Streptococcus mutans* agmatine deiminase system by low pH and agmatine. Appl Environ Microbiol 75:2629–2637. doi:10.1128/AEM.02145-08. 19270124 PMC2681689

[55] LeBlanc DJ, Lee LN, Abu-Al-Jaibat A 1992 Molecular, genetic, and functional analysis of the basic replicon of pVA380-1, a plasmid of oral streptococcal origin. Plasmid 28:130–145. doi:10.1016/0147-619X(92)90044-B. 1409970

[56] Markvicheva KN, Bogdanova EA, Staroverov DB, Lukyanov S, Belousov VV 2008 Imaging of intracellular hydrogen peroxide production with HyPer upon stimulation of HeLa cells with epidermal growth factor. Methods Mol Biol 476:79–86. doi:10.1007/978-1-59745-129-1_6. 19157010

