# Supplementary figures and images for: Redox-Regulated Adaptation of Streptococcus oligofermentans to Hydrogen Peroxide Stress
Source: mSystems. 2020 Mar 17;5(2):e00006-20. doi: 10.1128/mSystems.00006-20 (PMC7380579; doi:10.1128/mSystems.00006-20)

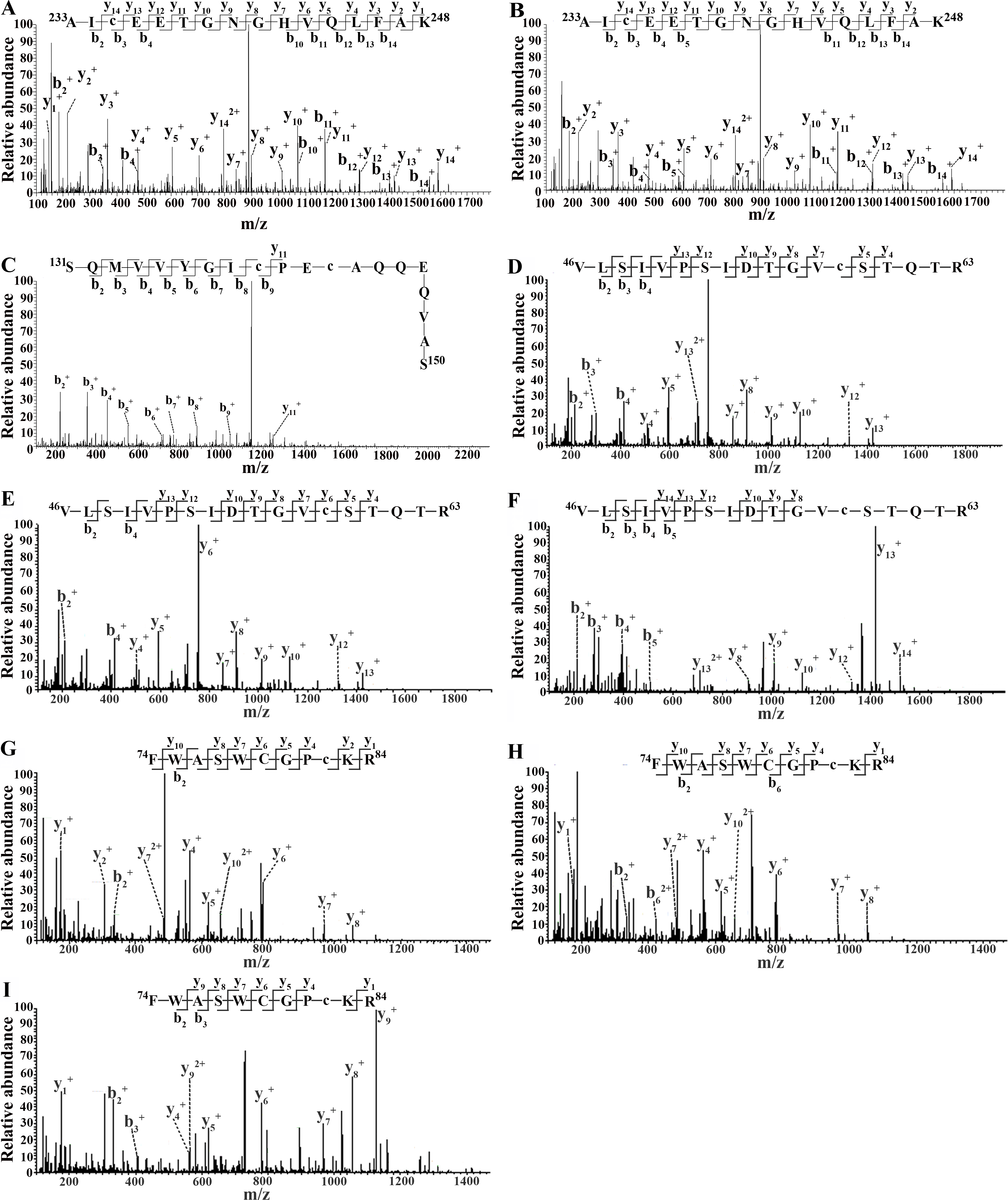

Supplement: FIG S1 [file mSystems.00006-20-sf001.tif]

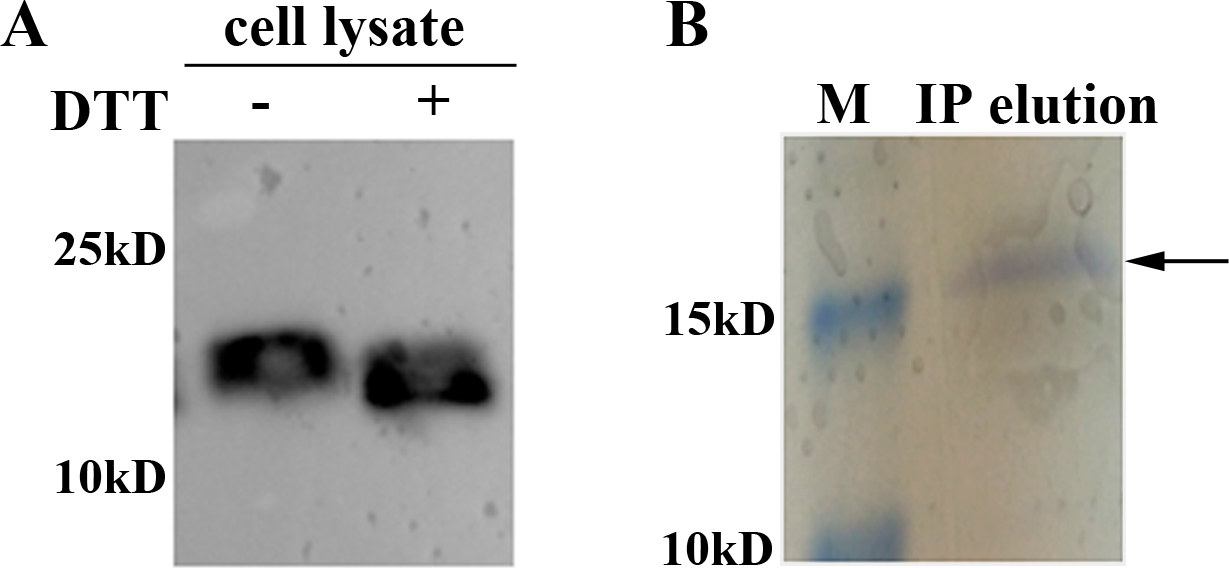

Supplement: FIG S2 [file mSystems.00006-20-sf002.tif]

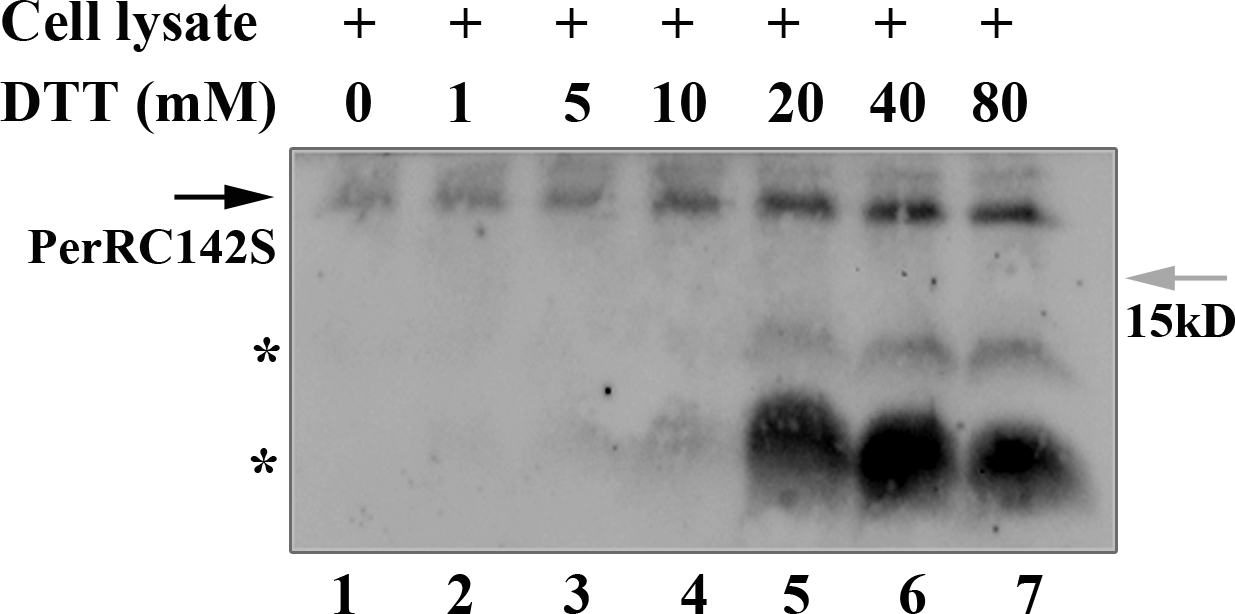

Supplement: FIG S3 [file mSystems.00006-20-sf003.tif]

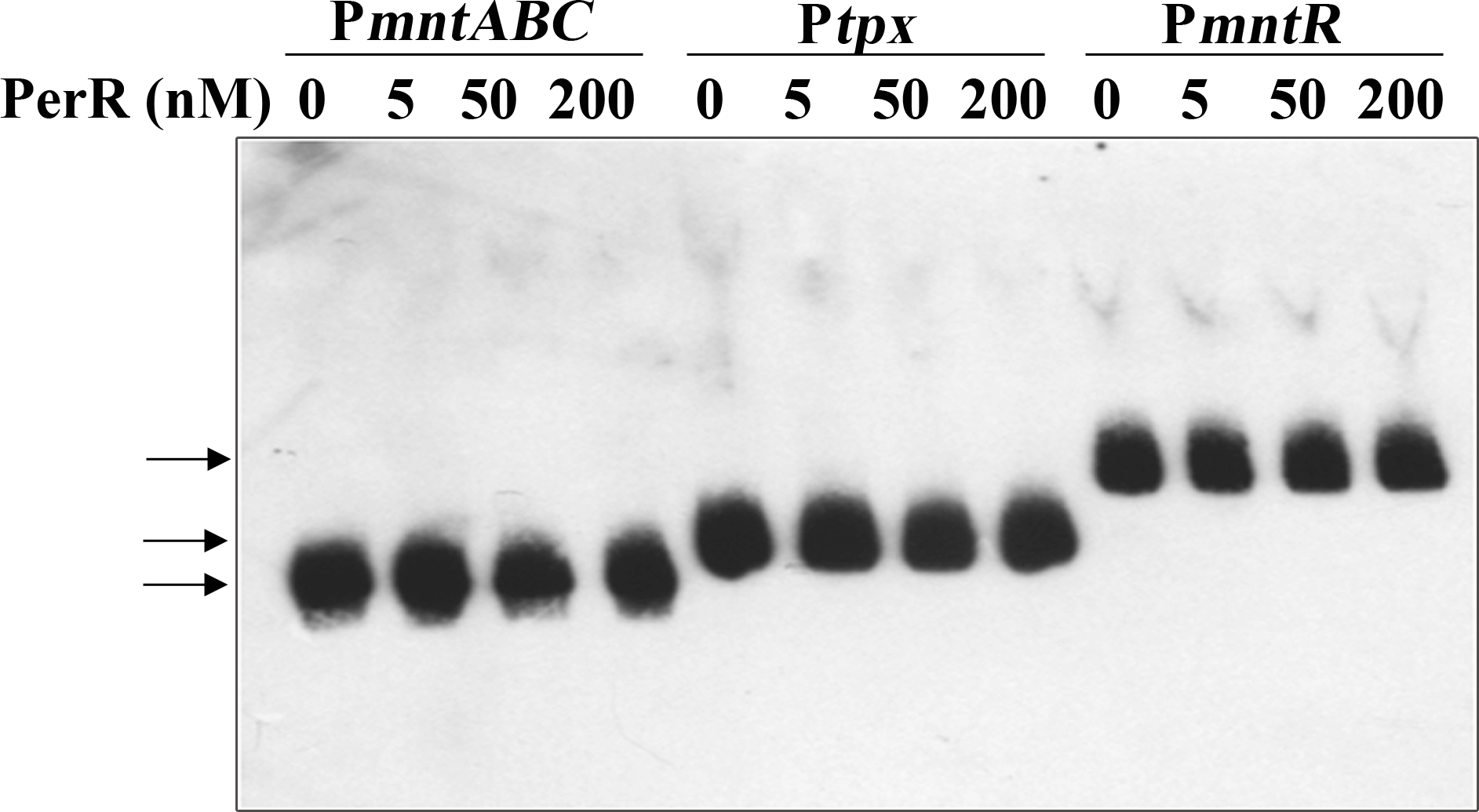

Supplement: FIG S4 [file mSystems.00006-20-sf004.tif]

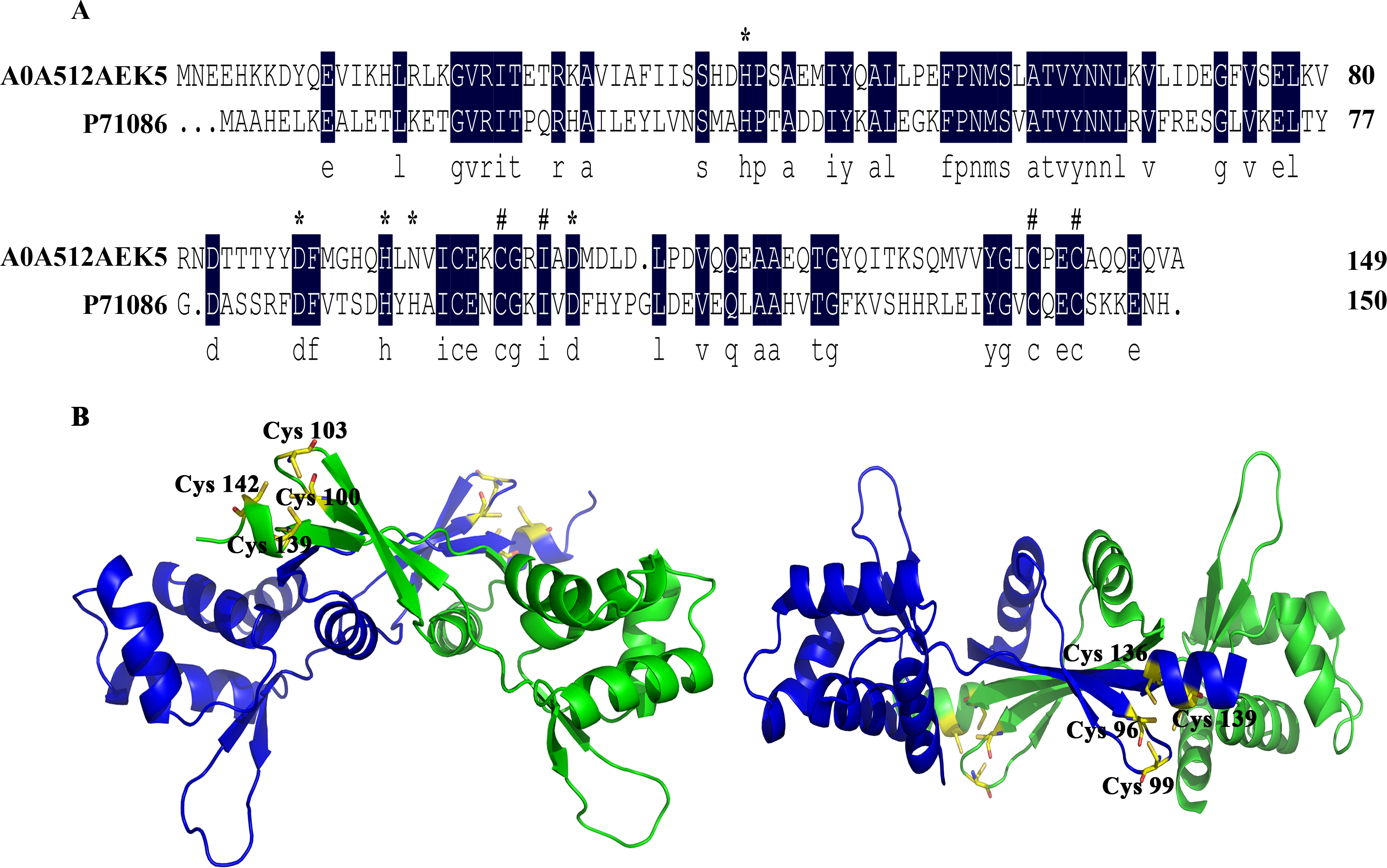

Supplement: FIG S5 [file mSystems.00006-20-sf005.tif]
