# Supplementary material for: Redox-Regulated Adaptation of Streptococcus oligofermentans to Hydrogen Peroxide Stress
Source: mSystems. 2020 Mar 17;5(2):e00006-20. doi: 10.1128/mSystems.00006-20 (PMC7380579; doi:10.1128/mSystems.00006-20)
Supplement: TABLE S1 [file mSystems.00006-20-st001.docx]

Table S1. Calculation of the oxidization ratio of cysteine or histidine residues in His6-tagged PerR that was immunoprecipitated from statically grown *S. oligofermentans*^a^

| Amino acid residues | Numbers of PSM fragments in oxidized state | Numbers of PSM fragments in reduced state | Oxidization ratio (%) | |
| --- | --- | --- | --- | --- |
| Cys100 | 328 | 103 | | 76 |
| Cys103 | 2 | 2 | | 50 |
| Cys139 | 478 | 98 | | 83 |
| Cys142 | 454 | 102 | | 82 |
| His5 | 1 | 13 | | 7 |
| His15 | 1 | 5 | | 17 |
| His38 | 0 | 320 | | 0 |
| His40 | 298 | 784 | | 28 |
| His93 | 57 | 357 | | 14 |
| His95 | 213 | 191 | | 53 |

^a^, Numbers of LC-MS/MS identified peptide spectral matches (PSMs) of the fragments containing targeting amino acid residues were counted and the oxidization ratios of the respective residues were calculated by dividing the number of oxidized PSMs s over the total PSMs.
