# Supplementary material for: Redox-Regulated Adaptation of Streptococcus oligofermentans to Hydrogen Peroxide Stress
Source: mSystems. 2020 Mar 17;5(2):e00006-20. doi: 10.1128/mSystems.00006-20 (PMC7380579; doi:10.1128/mSystems.00006-20)
Supplement: TABLE S2 [file mSystems.00006-20-st002.docx]

|  | Before H_2_O_2_ treatment | | | After H_2_O_2_ treatment | | |
| --- | --- | --- | --- | --- | --- | --- |
| Amino acid residues | Numbers of PSM fragments in reduced state | Numbers of PSM fragments in oxidized state | Oxidization ratio (%) | Numbers of PSM fragments in reduced state | Numbers of PSM fragments in oxidized state | Oxidization ratio (%) |
| His5 | 223 | 8 | 3 | 238 | 21 | 8 |
| His15 | 11 | 41 | 79 | 9 | 20 | 69 |
| His38 | 0 | 7 | 100 | 2 | 3 | 60 |
| His40 | 3 | 2 | 40 | 2 | 2 | 50 |
| His93 | 22 | 9 | 29 | 28 | 3 | 10 |
| His95 | 15 | 16 | 52 | 9 | 23 | 72 |

Table S2. Calculation of the H_2_O_2_-resulted histidine oxidation in the recombinant PerR:Zn,Mn protein^a^

^a^, Numbers of LC-MS/MS identified peptide spectral matches (PSMs) of the fragments containing histidine residues were counted and the oxidization ratios of histidine residues were calculated by dividing the number of oxidized PSMs over the total PSMs.
