# Supplementary material for: Redox-Regulated Adaptation of Streptococcus oligofermentans to Hydrogen Peroxide Stress
Source: mSystems. 2020 Mar 17;5(2):e00006-20. doi: 10.1128/mSystems.00006-20 (PMC7380579; doi:10.1128/mSystems.00006-20)
Supplement: TABLE S3 [file mSystems.00006-20-st003.docx]

Table S3. Strains, plasmids and primers used in this study^*^

| **Strain or plasmid** | **Characteristics and description** | | **References^a^ or source** |
| --- | --- | --- | --- |
| *E. coli* |  | |  |
| DH5α | *supE44 lacU169* (80 *lacZ*M15) *hsdR17 recA1 endA1 gyrA96 thi-1 relA1 luxS* | | TransGen Biotech, Beijing, China |
| *S. oligofermentans* |  | |  |
| Wild-type strain | AS 1.3089; Kan^s^ Sp^s^ | | 1 |
| WT-HyPer | AS 1.3089 pDL278-P*ldh*-HyPer; Sp^r^; AS 1.3089 with HyPer gene ectopically expressed | | 2 |
| ∆*mntR* | AS 1.3089 *mntR*::Kan; Kan^r^; AS 1.3089 with *mntR* deletion | | 3 |
| ∆*perR* | AS 1.3089 *perR*::Kan; Kan^r^; AS 1.3089 with *perR* deletion | | 4 |
| ∆*perR*/*mntR* | AS 1.3089 *perR*::Sp; *mntR*::Kan; Sp^r^; Kan^r^; AS 1.3089 with *perR mntR* double deletion | | This study |
| ∆*tpx* | AS 1.3089 *tpx*::Kan; Kan^r^; AS 1.3089 with *tpx* deletion | | This study |
| ∆*trx* | AS 1.3089 *trx*::Kan; Kan^r^; AS 1.3089 with *trx* deletion | | This study |
| ∆*dpr* | AS 1.3089 *dpr*::Kan; Kan^r^; AS 1.3089 with *dpr* deletion | | This study |
| Tpx-6His | AS 1.3089 Tpx::6His; Kan^r^; AS 1.3089 with Tpx carrying C-terminal 6×His tag | | This study |
| Trx-6His | AS 1.3089 Trx::6His; Kan^r^; AS 1.3089 with Trx carrying C-terminal 6×His tag | | This study |
| PerR-6His | AS 1.3089 PerR::6His; Kan^r^; AS 1.3089 with PerR carrying C-terminal 6×His tag | | This study |
| *perR*::pDL278-*perR*-6His | AS 1.3089 *perR*::Kan; pDL278-*perR*-6His; Kan^r^; Sp^r^; AS 1.3089 *perR* deletion strain carrying  *perR*-6His | | This study |
| *perR*::pDL278-*perR*C139S-6His | AS 1.3089 *perR*::Kan; pDL278-*perR*C139S-6His; Kan^r^; Sp^r^; AS 1.3089 *perR* deletion strain carrying  *perR*C139S-6His | | This study |
| *perR*::pDL278-*perR*C142S-6His | AS 1.3089 *perR*::Kan; pDL278-*perR*C142S-6His; Kan^r^; Sp^r^; AS 1.3089 *perR* deletion strain carrying  *perR*C142S-6His | | This study |
|  |  | | This study |
| **plasmid** |  | |  |
| pGEX-4T-1 | Amp^r^ | | GE, Boston, MA |
| pGEX-*perR* | pGEX-4T-1 carrying *perR*; Amp^r^ | | This study |
| pALH124 | Kan^r^ | | 5 |
| pDL278 | Sp^r^ | | 6 |
| pDL278-*perR*-6His | pDL278 carrying *perR*-6His; Sp^r^ | This study | |
| pDL278-*perR*C139S-6His | pDL278 carrying *perR*C139S-6His; Sp^r^ | This study | |
| pDL278-*perR*C142S-6His | pDL278 carrying *perR*C142S-6His; Sp^r^ | This study | |
| **Primer** | **Sequence (5’-3’)** | **Purpose** | |
| *tpx*upF | CGTTTGAGCGAAAAAGACCCAG | *tpx* deletion | |
| *tpx*upRBamHI | ATAT*GGATCC*GATTTCCGAGAAAGGTTGTC | *tpx* deletion | |
| *tpx*dnFBamHI | ATAT*GGATCC*CATTGCGGCAGTCAAAAATC | *tpx* deletion | |
| *tpx*dnR | CAATCCATGAAGTCTGGTCAGG | *tpx* deletion | |
| *trx*upF | AGAAAGATCGCTTGGACTGGTC | *trx* deletion | |
| *trx*upRBamHI | ATAT*GGATCC*GCTATCGAAGCAACAACTAG | *trx* deletion | |
| *trx*dnFBamHI | ATAT*GGATCC*CAGCAACGAAGAAGCAGAAAAAG | *trx* deletion | |
| *trx*dnR | TCGTTCCAGGTTCACCAGCAG | *trx* deletion | |
| *dpr*upF | GAAGGCTAGA AGTCCCAGAG | *dpr*deletion | |
| *dpr*upRBamHI | ATAT*GGATCC*CCATGTTTTACTTGAGTCAT | *dpr* deletion | |
| *dpr*dnFBamHI | ATAT*GGATCC*GGTCAAGCACCAGGTTTG | *dpr* deletion | |
| *dpr*dnR | CTATTTATCACCAGTTTTAGATGGC | *dpr* deletion | |
| *perR*upF | GATACTGAAGGCTAGAGAGG | *perR*deletion | |
| *perR*upRBamHI | AT*GGATCC*GGTAATCTTTTTTATGCTC | *perR*deletion | |
| *perR*dnFBamHI | AA*GGATCC*AGCAAGAACAAGTGGCTAG | *perR*deletion | |
| *perR*dnR | CTCATCGTCACGATCCATGTTTG | *perR*deletion | |
| Tpx6HisupF | GGCGGCATCCAAATTCGGGTGTCG | Construction of Tpx-6His strain | |
| Tpx6HisupRBamHI | AT*GGATCC*TTAGTGGTGGTGGTGGTGGTGTAGATTTTTGACTGCCGC | Construction of Tpx-6His strain | |
| Tpx6HisdnFBamHI | AT*GGATCC*AGAAACATCTTTTTCAACAAAATGC | Construction of Tpx-6His strain | |
| Tpx6HisdnR | GGCTGACGCCATTTCAAATGC | Construction of Tpx-6His strain | |
| Trx6HisupF | GGACGCCTTGTATTGGCCCTGTCC | Construction of Trx-6His strain | |
| Trx6HisupRBamHI | AT*GGATCC*TTAGTGGTGGTGGTGGTGGTGTTTCATTTCTTTAAAAGC | Construction of Trx-6His strain | |
| Trx6HisdnFBamHI | AT*GGATCC*AGTAAAAATAGAGTCGGACAG | Construction of Trx-6His strain | |
| Trx6HisdnR | GAACGTAGAATGGGTAGTTGGTG | Construction of Trx-6His strain | |
| PerR6HisupF | CCTGCAGGGCAGGTAAAATAGCAGAAGATTG | Construction of PerR-6His strain | |
| PerR6HisupRBamHI | TA*GGATCC*CTAGTGGTGGTGGTGGTGGTGGCTAGCCACTTGTTC | Construction of PerR-6His strain | |
| PerR6HisdnFBamHI | CCGC*GGATCC*AAGTCTTGAGTGAGAATTAATTTTC | Construction of PerR-6His strain | |
| PerR6HisdnR | GGAAGGGCACGTTCCAAAGTTACTTTC | Construction of PerR-6His strain | |
| PerR6HiscomFEcoRI | AA*GAATTC*GTGATAGAAAACCAGCTG | For PerR6His complement | |
| PerR6HiscomRSalI | ATAT*GTCGAC*CTAGTGGTGGTGGTGGTGGTGGCTAGCCACTTGTTCTTG | For PerR6His complement | |
| perR6HisC139SF | GGTCGTTTATGGCATTAGTCCAGAGTGTGCCCAG | For PerRC139S6His complement | |
| perR6HisC139SR | CTGGGCACACTCTGGACTAATGCCATAAACGACC | For PerRC139S6His complement | |
| perR6HisC142SF | GGCATTTGTCCAGAGAGTGCCCAGCAAGAACAA | For PerRC142S6His complement | |
| perR6HisC142SR | TTGTTCTTGCTGGGCACTCTCTGGACAAATGCC | For PerRC142S6His complement | |
| PerR-GSTEcoRI-F | AAAT*GAATTC*ATGAATGAGGAGCATAAAAAAG | Recombinant PerR-GST expression | |
| PerR-GSTXhoI-R | AAAT*CTCGAG*CTAGCTAGCCACTTGT | Recombinant PerR-GST expression | |
| Gi09640F | CTTTAAGAAGGAGATATACCATGACAACCTTTCTCGGAAATC | Recombinant Tpx-6His expression | |
| Gi09640R | CAGTGGTGGTGGTGGTGGTGTAGATTTTTGACTGCCGCAATG | Recombinant Tpx-6His expression | |
| Gi03205F | CTTTAAGAAGGAGATATACCATGAAAAAAATTCTTTCGCTAG | Recombinant Trx-6His expression | |
| Gi03205R | CAGTGGTGGTGGTGGTGGTGTTTCATTTCTTTAAAAGCTTTTTC | Recombinant Trx-6His expression | |
| pET28aF | GGTATATCTCCTTCTTAAAGTTAAACAAAATTATTTCTAGAGGGGAATTGTTATC | Recombinant Trx-6His expression | |
| pET28aR | CACCACCACCACCACCACTG | Recombinant Trx-6His expression | |
| PdprEMSAF | Biotin-AATGACCAAGCCTAGAAAT | For PerR EMSA | |
| PdprEMSAR | AATGATACCTCTCTTTTTATTTGT | For PerR EMSA | |
| PmntAEMSAF | Biotin-ATCTGGGATAACCGCTGTTTTG | For PerR EMSA | |
| PmntAEMSAR | CATTTCAATCATTGTTACACCTCTTTTG | For PerR EMSA | |
| PtpxEMSAF | Biotin-AAAGACACTAACATCCCAATCTAC | For PerR EMSA | |
| PtpxEMSAR | CGAGAAAGGTTGTCATTAAAAGTCTC | For PerR EMSA | |
| PmntREMSAF | Biotin-ATAGCTTCTGAAAGAGGGGACTG | For PerR EMSA | |
| PmntREMSAR | CTTTATTTGGCGTCATTTTCAGTCCTCG | For PerR EMSA | |
| *mntR*RTF | GTGTCCACGATAACTTTGAG | *mntR* qPCR | |
| *mntR*RTR | CAATCATAGGGCTGACTTGA | *mntR* qPCR | |
| *mntA*RTF | ACATGAATACGAGCCTCTGCCTG | *mntA* qPCR | |
| *mntA*RTR | GCCTTCCAAGTAAATCACGTCC | *mntA* qPCR | |
| *dpr*RTF | GAAGATTTCAATCACGCGCTCCAA | *dpr* qPCR | |
| *dpr*RTR | TTATACAGCTCGTATCGCTCTT | *dpr* qPCR | |
| *tpx*RTF | TGTTATGCTATCAGACTACT | *tpx* qPCR | |
| *tpx*RTR | GGCTCAGTATTGATATTGTC | *tpx* qPCR | |
| *trx*RTF | GTCCAATCGCTGGTTGCAGTTG | *trx* qPCR | |
| *trx*RTR | CCTTGGAGACCCGGTGCTAC | *trx* qPCR | |
| 16SRTF | CTGTTGTAAGAGAAGAACGAGTG | 16S rDNA qPCR | |
| 16SRTR | CCACAGCCTTTAACTTCAGACTTATC | 16S rDNA qPCR | |

^*^: Amp, ampicillin; Kan, kanamycin; Sp, spectinomycin; r, resistant; s, sensitive;

Italic nucleotide bases indicate restriction enzyme digestion sites.

**^a^ References**

1. Tong H, Gao X, Dong X. 2003. *Streptococcus oligofermentans* sp. nov., a novel oral isolate from caries-free humans. Int J Syst Evol Microbiol 53:1101-4.

2. Tong H, Wang X, Dong Y, Hu Q, Zhao Z, Zhu Y, Dong L, Bai F, Dong X. 2019. A *Streptococcus* aquaporin acts as peroxiporin for efflux of cellular hydrogen peroxide and alleviation of oxidative stress. J Biol Chem 294:4583-4595.

3. Chen Z, Wang X, Yang F, Hu Q, Tong H, Dong X. 2017. Molecular insights into hydrogen peroxide-sensing mechanism of the metalloregulator MntR in controlling bacterial resistance to oxidative sresses. J Biol Chem 292:5519-5531.

4. Wang X, Tong H, Dong X. 2014. PerR-regulated manganese ion uptake contributes to oxidative stress defense in an oral streptococcus. Appl Environ Microbiol 80:2351-9.

5. Liu Y, Zeng L, Burne RA. 2009. AguR is required for induction of the *Streptococcus mutans* agmatine deiminase system by low pH and agmatine. Appl Environ Microbiol 75:2629-37.

6. LeBlanc DJ, Lee LN, Abu-Al-Jaibat A. 1992. Molecular, genetic, and functional analysis of the basic replicon of pVA380-1, a plasmid of oral streptococcal origin. Plasmid 28:130-45.
